# Effectiveness and cost-effectiveness of eliminating cervical cancer through a tailored optimal pathway: a modeling study

**DOI:** 10.1186/s12916-021-01930-9

**Published:** 2021-03-03

**Authors:** Changfa Xia, Xiaoqian Xu, Xuelian Zhao, Shangying Hu, Youlin Qiao, Yong Zhang, Raymond Hutubessy, Partha Basu, Nathalie Broutet, Mark Jit, Fanghui Zhao

**Affiliations:** 1 National Cancer Center/National Clinical Research Center for Cancer/Cancer Hospital, Chinese Academy of Medical Sciences and Peking Union Medical College, No.17 Pan-jia-yuan South Lane, Chaoyang District, Beijing, 100021 China; 2 Department of Immunization, Vaccines and Biologicals (IVB), World Health Organization, Geneva, Switzerland; 3 Screening Group, Early Detection and Prevention Section, International Agency for Research on Cancer, Lyon, France; 4 Department of Reproductive Health and Research - WHO Special Research Programme on Human Reproduction (HRP), World Health Organization, Geneva, Switzerland; 5 Department of Infectious Disease Epidemiology, London School of Hygiene & Tropical Medicine, London, UK; 6 School of Public Health, University of Hong Kong, Hong Kong, Hong Kong SAR, China

**Keywords:** Cervical cancer, Elimination, Optimal pathway, Cost-effectiveness

## Abstract

**Background:**

The World Health Assembly has adopted a global strategy to eliminate cervical cancer. However, neither the optimal pathway nor the corresponding economic and health benefits have been evaluated. We take China as an example to assess the optimal pathway towards elimination and the cost-effectiveness of tailored actions.

**Methods:**

A validated hybrid model was used to assess the costs and benefits of alternative strategies combining human papillomavirus vaccination, cervical screening, and treatment of pre-invasive lesions and invasive cancer for females with different immunization history. All Chinese females living or projected to be born during 2015–2100, under projected trends in aging, urbanization, and sexual activity, were considered. Optimal strategies were determined by cost-effectiveness efficiency frontiers. Primary outcomes were cervical cancer cases and deaths averted and incremental cost-effectiveness ratios (ICERs). We employed a lifetime horizon from a societal perspective. One-way and probabilistic sensitivity analyses evaluate model uncertainty.

**Results:**

The optimal pathway represents an integration of multiple tailored strategies from females with different immunization history. If China adopts the optimal pathway, the age-standardized incidence of cervical cancer is predicted to decrease to fewer than four new cases per 100,000 women (i.e., elimination) by 2047 (95% confidence interval 2043 to 2050). Compared to the status quo, the optimal pathway would avert a total of 7,509,192 (6,922,744 to 8,359,074) cervical cancer cases and 2,529,873 (2,366,826 to 2,802,604) cervical cancer deaths in 2021–2100, with the discounted ICER being $− 339 (− 687 to − 79) per quality-adjusted life-year.

**Conclusions:**

By adopting an optimal pathway from 2021 (namely, the year of the first Chinese Centennial Goals) onwards, cervical cancer could be eliminated by the late 2040s (namely, ahead of the second Chinese Centennial Goals) while saving net economic costs in China.

**Supplementary Information:**

The online version contains supplementary material available at 10.1186/s12916-021-01930-9.

## Background

Following the call from the World Health Organization (WHO)’s Director-General for action towards the elimination of cervical cancer as a public health problem, several modeling studies suggested that the goal of elimination is within reach globally in the twenty-first century [1–3]. All of these studies were designed to evaluate the time frame and intervention strategies needed to achieve cervical cancer elimination or to predict the impact on cervical cancer mortality and deaths averted [1–3]. In August 2020, the World Health Assembly has adopted the global strategy to accelerate the elimination of cervical cancer. However, to the best of our knowledge, a full economic evaluation of a comprehensive cervical cancer elimination strategy has yet to be performed.

China contributed to 20.2% of the global burden of cervical cancer, and the incidence showed a significant rise of up to 10.5% per year [4]. The mean age of cervical cancer patients was about 53 years, and 57.6% of patients were diagnosed as stage I [4, 5]. HPV prevalence in cervical cancer patients was 97.6% in China, with HPV-16 and HPV-18 accounting for 59.5 and 9.6%, respectively [6]. However, China has not yet included human papillomavirus (HPV) vaccination into its national immunization program (NIP), and the 3-year coverage rate of cervical screening in women aged 35–64 years was 22.6% [7, 8]. At the end of 2019, China’s first domestically produced bivalent HPV vaccine (Cecolin™) was approved for cervical cancer prevention in the female population aged 9–45 years [9]. The 5.5-year follow-up suggested that the vaccine is highly efficacious in preventing genital lesions and persistent infection associated with HPV-16/18, with no waning protection detected after vaccination [10, 11]. In 2020, the issue of introducing HPV vaccination into the NIP was submitted to the Chinese government for consideration [12]. In the absence of evidence-based analyses on the impact of an optimal strategy involving both HPV vaccination and cervical cancer screening, policy-makers in China are still hesitant to make major changes.

Although there is great optimism about eliminating cervical cancer in China [13], the optimal pathway considering the availability of healthcare resources and health equity has not been established. Therefore, from the perspective of public health investment, a full health economic evaluation needs to be carried out. Our present study assesses the optimal pathway towards cervical cancer elimination in the twenty-first century in China, evaluates the economic investment needed to adopt the proposed activities, and discusses its significance for public health.

## Methods

The results were reported following the Consolidated Health Economic Evaluation Reporting Standards Statement (CHEERS) (Additional file 1: CHEERS checklist).

In this model-based economic evaluation, we assessed the effect of several HPV vaccination and cervical screening scenarios on cervical cancer elimination in China under projected aging, urbanization, and sexual activity trends. All Chinese women living or projected to be born during 2015–2100 were considered to obtain estimates of costs and health outcomes of all strategies. The population was stratified by area of residence (urban and rural), gender, sexual activity (high, low, none), and age group (0–84 per year, and 85+).

### Mathematical model

We updated the hybrid model which was developed to evaluate cervical cancer prevention strategies in China [14]. Please see Additional file 2 for more details on the model (Additional file 2: Table S1–S6 and Figure S1–S2) [2, 3, 6, 7, 9, 11, 12, 14–49]. We incorporated 13 HPV strains into our analyses and modeled the impact of every high-risk HPV strain separately [50]. The number of women in each disease state, year, risk group, HPV type (if infected), and age estimated from a dynamic transmission model was fed into the natural history model to simulate the costs and outcomes of each strategy.

Models were calibrated using epidemiological data of high-risk HPV prevalence, cervical incidence and mortality in 2015, HPV type distributions in women with normal cervical cytology, low-grade cervical precancerous lesions, high-grade cervical precancerous lesions, and invasive cervical cancer (Additional file 2: Table S3–S6 and Figure S2) [6, 47–49, 51].

### Inputs and assumptions

Base-case estimates and ranges for all parameters are listed in tables in the supplementary file (Additional file 2: Table S2) [2, 3, 14, 19, 26, 28, 30–45]. We assumed that infection-acquired immunity would wane over time, whereas vaccine-acquired immunity would be lifelong [2, 3, 40, 52]. For the base-case analyses, HPV vaccination was assumed to provide full efficacy against vaccine-targeted HPV types, while the cross-protection against non-vaccine targeted types was not considered. The screening algorithm selected for modeling involved primary screening with the high-risk HPV test and triaging of the HPV-positive women with liquid-based cytology (LBC). The sensitivity of HPV DNA testing was assumed to be 90% for cervical intraepithelial neoplasia (CIN) grade 2, 94% for CIN3, and 100% for invasive cervical cancer. Age-specific cervical cancer survival rates were extracted from population-based cancer registries in urban and rural China [53].

We derived utility values that were specific for patients’ health and treatment state from our hospital-based prospective study in China [44]. Health-related quality of life for patients undergoing treatment for CIN1 and those receiving terminal care was based on internationally published data [43].

Costs of domestically manufactured bivalent HPV vaccine and 9-valent HPV vaccine in the NIP were assumed to be $4.6 and $6.6 per dose, respectively, and the vaccine administration cost was estimated at $3.83 per dose (Additional file 2: Table S2) [7, 9, 11, 12, 15–20]. Cervical screening costs using LBC and HPV DNA testing were estimated at $9.46 and $15.13 per screen in urban areas, and $7.00 and $12.98 per screen in rural areas, respectively (Additional file 2: Table S1) [6, 21–27, 29]. We considered all components of direct medical cost, direct non-medical costs, and indirect costs for women diagnosed with CIN and invasive cervical cancer. All unit costs were adjusted to the year 2020 using the government-reported consumer price index for health care [51] and then converted into US dollar using exchange rates for early 2020 (i.e., 1.00 US dollar = 7.00 Chinese yuan).

### Alternative scenarios

Considering the progress of HPV vaccine research and development in China, the domestically manufactured bivalent HPV vaccine is the only product that will be considered for inclusion in the NIP until 2030; after that year, domestically manufactured low-cost 9-valent HPV vaccine will also be considered [54]. HPV vaccination is assumed to be delivered to girls aged 9 years with two dose regimens routinely and catch-up to 14 years (two dose regimens) or to 25 years (three dose regimens for girls aged 15 and older) [10]. HPV-based cervical screening is assumed to be targeted at women aged 35–64 years. We considered six alternative screening strategies for unvaccinated women and 23 alternative strategies for vaccinated women (Additional file 2: Figure S3). Currently, about 45% of women diagnosed with CIN1 are treated in China (Additional file 2: Table S2) [14]. We assume that the practice will change from 2031, with women with CIN1 undergoing two follow-up visits before being treated.

We assumed the maximum achievable coverage of routine vaccination and catch-up vaccination would be 95% and 90%, respectively, and that maximum achievable coverage of cervical screening would be 90% [14]. Due to shortages in HPV vaccine supply [55], we assume that China will vaccinate only 9-year-old girls in 2021, and catch-up vaccination would be scaled-up to girls/women aged 10–14 or 10–25 years in 2022–2025. The maximum capacity for cervical screening in China in 2021 is estimated to be 35 million women [8, 56]; therefore, if China screens women aged 35–64 every 3 years, the maximum achievable coverage would be 35% in 2021, while if China shifts the screening interval to 5 years, the maximum achievable coverage would be 60% in 2021. We assumed that screening coverage would scale up by 5% every year until it reaches 70%, following which the rate of increase would slow down to 3% every year till 90% coverage is achieved.

### Outcomes

We estimated the lifetime costs and health benefits for each strategy in comparison with the status quo strategy (3-yearly screening with coverage of 26.6% in urban areas and 19.3% in rural areas; HPV vaccination coverage is negligible) in China [14]. The cost of implementing each strategy was estimated from a societal perspective. The health outcomes of each strategy were evaluated in quality-adjusted life-years (QALYs), taking into account health state utility weights. Both costs and health outcomes were discounted at an annual rate of 3% [45].

In the base-case analysis, status quo strategy and optimal strategy were evaluated in the model to estimate the year of cervical cancer elimination, averted cervical cancer cases and deaths, and the incremental cost-effectiveness ratios (ICER) for urban and rural areas, respectively. The ICER was calculated as incremental cost per additional QALY gained between optimal strategy and status quo.

We calculated age-standardized annual incidence and mortality rates using Segi’s world standard population. Elimination of cervical cancer as a public health problem was defined as the first year when the age-standardized incidence is below 4/100,000 women.

### Main analysis

Using the calibrated model, we assessed the costs and benefits of all alternative scenarios in birth cohorts with different HPV immunization history (unvaccinated, vaccinated with a bivalent vaccine, and vaccinated with a 9-valent vaccine), different target ages at vaccination and coverage, and different cervical screening characteristics (with different target ages, screening intervals, and coverage). A five-stage analytic process was involved to determine the optimal pathway (Additional file 2: Table S7–S8 and Figure S3). We selected the optimal strategies by determining the ICER as compared with the next-least-expensive strategy lying on the efficiency frontier (next-best strategy). We applied the Chinese median annual income ($3789) and gross domestic product (GDP) per capita ($10,276) as the threshold ICER when selecting the optimal strategies [57, 58].

### Sensitivity analysis

We varied each input value in the model over a plausible range in one-way deterministic sensitivity analyses to examine the impact of uncertainty in individual input parameters on the results. Probabilistic sensitivity analysis was conducted by performing 1000 Monte Carlo simulations to sample parameter values from their distributions and estimate outcomes. The results of the probabilistic sensitivity analysis were used to calculate the 95% confidence intervals (CIs) of model results.

## Results

**Fig. 1:**
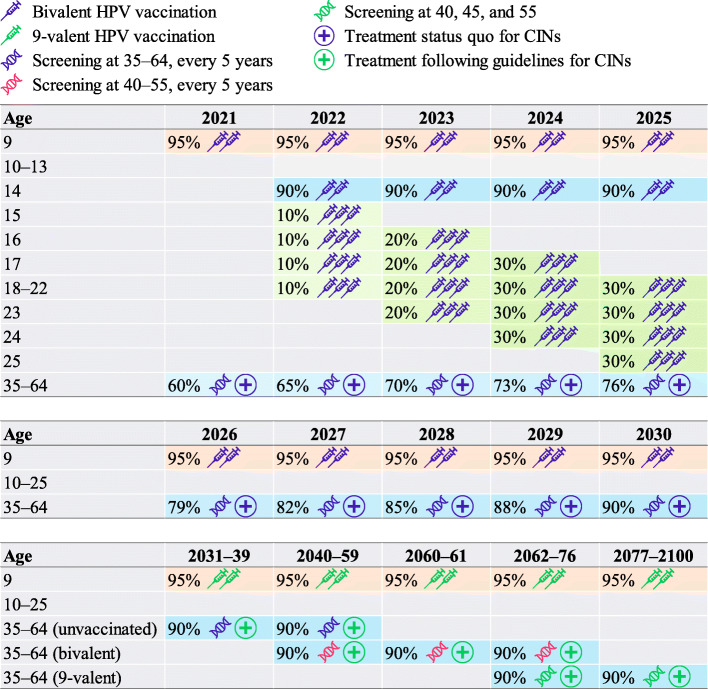
Optimal pathway towards cervical cancer elimination. The percentages and background colors in each cell indicate the population coverages. HPV, human papillomavirus; CIN, cervical intraepithelial neoplasia

The optimal pathway towards the elimination of cervical cancer in China is to integrate the tailored optimal strategies of different birth cohorts under the constraints of available resources (Fig. 1 and Additional file 2: Table S7–8 and Figure S3). Specifically, routine bivalent HPV vaccination should be initiated in 2021 for girls (at 95% expected coverage) aged 9 years old and switched to 9-valent vaccine in 2031 when it becomes available for the Chinese NIP. Catch-up vaccination should target girls (at 90% expected coverage) up to 25 years old in 2022–2025, with the most rapid scale-up possible under the expected vaccine supply constraints. HPV-based screening should be offered (at 90% expected coverage) to unvaccinated women aged 35–64 every 5 years, with the most rapid scale-up to the population possible under the constraints of screening service resources. For the bivalent vaccinated cohort, screening should target women aged 40–55 every 5 years, while for the 9-valent vaccinated cohort, screening at 40, 45, and 55 would be a cost-effective strategy while gaining the most benefits.

**Fig. 2:**
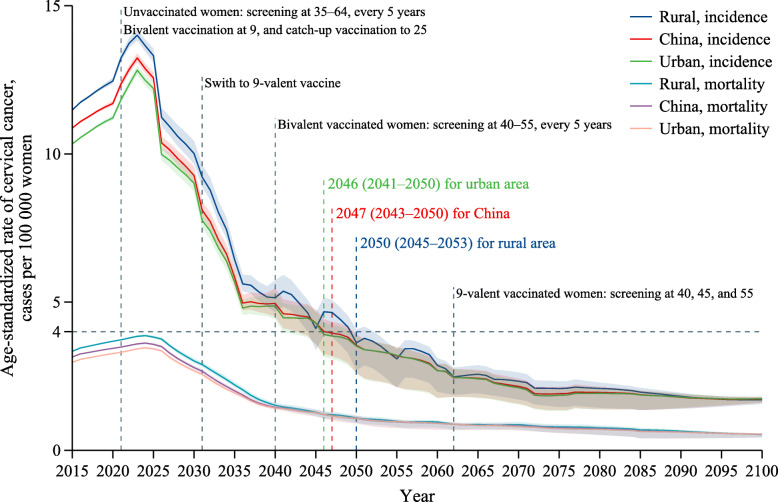
Age-standardized cervical cancer incidence and mortality for the optimal pathway. The solid line represents the base case estimates, and the shaded area represents the 95% confidence intervals based on 1000 simulations of probabilistic sensitivity analysis

The optimal pathway is predicted to lead to cervical cancer elimination in China by 2047 (95% CI, 2043 to 2050) (Fig. 2). This pathway is a cost-saving strategy with a discounted ICER of $− 339 (− 687 to − 79) per QALY compared to the status quo strategy (Table 1). A total of 7,509,192 (6,922,744 to 8,359,074) cervical cancer cases and 2,529,873 (2,366,826 to 2,802,604) cervical cancer deaths in 2021–2100 would further be averted in China by adopting the optimal pathway compared to the status quo (Table 2). Cervical cancer elimination can be achieved by 2046 (2041 to 2050) in urban areas and 2050 (2045 to 2053) in rural areas, with the discounted ICER being $− 511 (− 913 to − 199) per QALY in urban areas and $234 (− 120 to 544) per QALY in rural areas compared to the status quo strategy. We estimated that the optimal pathway would further averts 5,714,707 (5,227,933 to 6,387,465) and 1,794,485 (1,538,873 to 2,131,125) cervical cancer cases, and 1,939,440 (1,796,327 to 2,161,390) and 590,432 (507,330 to 702,435) cervical cancer deaths compared to the status quo in urban areas and rural areas, respectively.

**Table 1:** Lifetime costs, effectiveness, and incremental cost-effectiveness for optimal pathway versus status quo

Scenario	0% discount rate			3% discount rate		
	Total costs (95% CI), million $	Total QALYs (95% CI), million	ICER (95% CI), $/QALY	Total costs (95% CI), million $	Total QALYs (95% CI), million	ICER (95% CI), $/QALY
China						
Status quo	235,233 (202,975 to 274,054)	62,061 (53,812 to 71,934)	–	64,516 (60,785 to 68,688)	19,907 (18,803 to 21,106)	–
Optimal pathway	135,523 (117,264 to 151,530)	62,177 (53,911 to 72,071)	− 861 (− 998 to − 762)	59,222 (53,732 to 63,311)	19,922 (18,818 to 21,122)	− 339 (− 687 to − 79)
Incremental value	− 99,711 (− 129,313 to − 79,441)	116 (98 to 138)	–	− 5295 (− 11,148 to − 1197)	16 (15 to 17)	–
Urban area						
Status quo	192,864 (167,384 to 224,928)	46,214 (40,370 to 53,409)	–	53,387 (49,723 to 57,458)	14,689 (13,864 to 15,656)	–
Optimal pathway	108,875 (93,177 to 123,739)	46,299 (40,442 to 53,509)	− 987 (− 1149 to − 858)	47,250 (42,273 to 51,218)	14,701 (13,876 to 15,669)	− 511 (− 913 to − 199)
Incremental value	− 83,990 (− 108,944 to − 65,382)	85 (73 to 101)	–	− 6137 (− 11,347 to − 2345)	12 (11 to 13)	–
Rural area						
Status quo	42,369 (33,919 to 53,818)	15,846 (13,269 to 19,448)	–	11,130 (9565 to 12,860)	5218 (4733 to 5818)	–
Optimal pathway	26,648 (21,842 to 31,565)	15,877 (13,294 to 19,488)	− 512 (− 666 to − 380)	11,972 (10,405 to 13,413)	5222 (4736 to 5822)	234 (− 120 to 544)
Incremental value	− 15,721 (− 23,542 to − 10,227)	31 (25 to 39)	–	842 (− 458 to 1919)	4 (3 to 4)	–

Components may not sum to totals due to rounding

*QALYs*, quality-adjusted life-years; *ICER*, incremental cost-effectiveness ratio

**Table 2:** Estimated cervical cancer cases and deaths averted for optimal pathway versus status quo

Period	Cervical cancer cases averted (95% CI), no.			Cervical cancer deaths averted (95% CI), no.		
	China	Urban area	Rural area	China	Urban area	Rural area
2021–2030	104,684 (92,294 to 117,425)	79,967 (69,927 to 89,751)	24,717 (20,495 to 28,791)	32,248 (30,575 to 34,004)	22,986 (21,566 to 24,365)	9262 (8572 to 10,025)
2031–2040	787,870 (755,292 to 821,308)	617,718 (585,630 to 650,763)	170,151 (150,964 to 188,778)	223,549 (215,113 to 232,653)	172,801 (164,115 to 181,471)	50,748 (45,154 to 56,232)
2041–2050	1,055,622 (1,010,812 to 1,118,157)	842,780 (800,376 to 897,473)	212,842 (182,616 to 247,162)	343,138 (330,470 to 358,224)	277,800 (264,190 to 292,840)	65,338 (55,339 to 75,919)
2051–2060	1,132,932 (1,092,152 to 1,219,614)	873,869 (832,217 to 951,946)	259,064 (227,593 to 299,560)	382,491 (369,713 to 402,859)	305,618 (291,910 to 325,951)	76,873 (65,333 to 89,969)
2061–2070	1,137,444 (1,075,807 to 1,227,010)	865,938 (809,346 to 942,036)	271,506 (237,146 to 313,962)	385,328 (369,061 to 408,807)	298,520 (281,708 to 319,957)	86,808 (74,878 to 100,401)
2071–2080	1,118,296 (1,020,789 to 1,256,136)	840,715 (759,702 to 943,881)	277,581 (240,072 to 330,623)	379,921 (352,929 to 418,315)	283,755 (260,544 to 313,656)	96,165 (83,562 to 112,677)
2081–2090	1,091,456 (966,871 to 1,291,902)	804,785 (706,821 to 953,063)	286,671 (242,893 to 353,727)	391,759 (353,916 to 465,217)	288,997 (258,995 to 344,650)	102,762 (88,340 to 127,582)
2091–2100	1,080,888 (908,727 to 1,307,522)	788,935 (663,914 to 958,552)	291,953 (237,094 to 368,522)	391,439 (345,049 to 482,525)	288,963 (253,299 to 358,500)	102,476 (86,152 to 129,630)
Total	7,509,192 (6,922,744 to 8,359,074)	5,714,707 (5,227,933 to 6,387,465)	1,794,485 (1,538,873 to 2,131,125)	2,529,873 (2,366,826 to 2,802,604)	1,939,440 (1,796,327 to 2,161,390)	590,432 (507,330 to 702,435)

Components may not sum to totals due to rounding

**Fig. 3:**
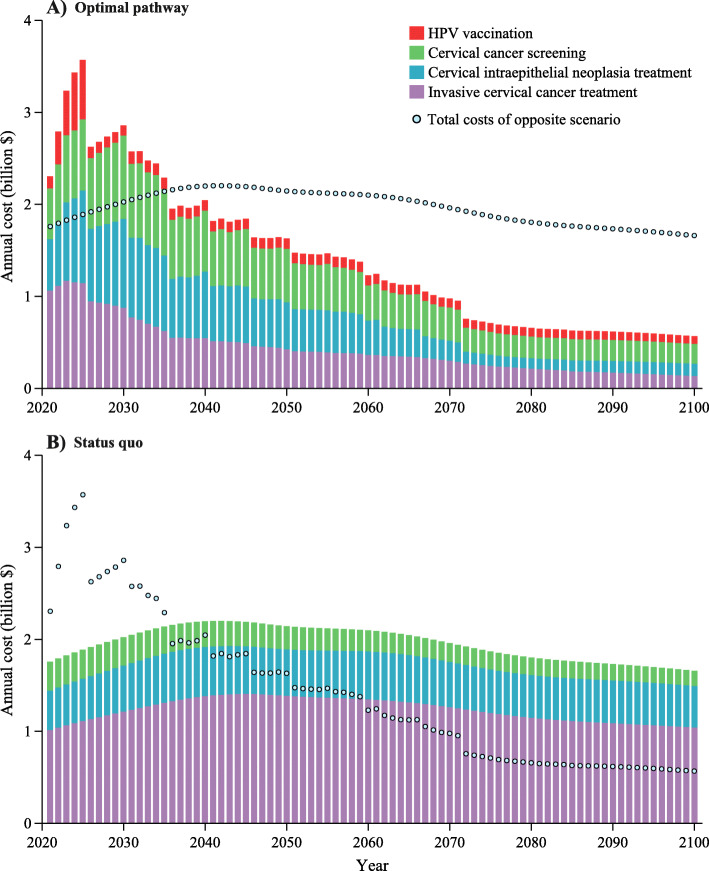
Annual costs from each component of cervical cancer prevention and treatment. Total costs and cost components for scenarios of the optimal pathway (**a**) and status quo (**b**)

Net costs would be positive in 2021–2035 by adopting the optimal pathway, while from 2036 onwards, annual net costs would be negative, due to reductions in the cost of invasive cervical cancer treatment (Fig. 3 and Additional file 2: Figure S6–S7). Annual benefits (i.e., incremental QALYs) of the optimal pathway would be slightly lower than that of the status quo before 2026 because of the lead time from cervical screening, while it would be much higher from 2027 onwards (Additional file 2: Figure S8). More intensive screening strategies would be considered cost-effective when using the GDP per capita as the threshold (Additional file 2: Figure S9). The year of cervical cancer elimination would thus be 11 years ahead of that using median income as the threshold (Additional file 2: Figure S10). However, the ICER would be $4344 (3357 to 6056) per QALY for the optimal pathway using GDP per capita as the threshold compared to the optimal pathway using the median income as the threshold (Additional file 2: Table S9–S11 and Figure S11–S12).

The uncertainty ranges of individual variables had a small-to-moderate effect on cost-effectiveness (ICER range, $− 861 to $474 per QALY) (Additional file 2: Figure S4–S5). The model was most sensitive to the discount rate, the clearance rate of HPV infection, screening cost, transition probability, and cross-protect of the bivalent vaccine. Input to which the results were most sensitive was discount rate both in urban and rural areas, which potentially increased the ICER to $219 per QALY in urban areas and $1372 per QALY in rural areas.

## Discussion

We have conducted, for the first time, a health economic evaluation to determine the optimal pathway and assess the costs and benefits of strategies for cervical cancer elimination worldwide. We estimated that cervical cancer could be eliminated as a public health problem by the late 2040s in a cost-saving manner by adopting the optimal pathway. Compared to the status quo, the optimal pathway represents a saving of about $100 billion of costs and 116 million of QALYs in women living or projected to born during 2021–2100 and averts about 7.5 million cervical cancer cases and 2.5 million cervical cancer deaths in 2021–2100 in China.

Our previous study has quantified the impact of introducing bivalent HPV vaccine into the NIP on cervical cancer elimination in China but did not provide economic evidence [14]. In this study, we have addressed the major limitations of our previous study by outlining the optimal cervical cancer control pathway from the perspective of cost-effectiveness [13, 14]. This study builds on our previous work in several ways. First, we used an age-structured population rather than a single cohort to select the optimal strategies [13]. Second, we considered all components of costs and employed QALYs as the primary outcome, rather than only considered costs from the government and used the year of elimination as the primary outcome. As a straightforward health economic evaluation, QALYs adopted in this study are more adequate to quantify the optimal strategy of cervical cancer elimination, because the earliest time of elimination is not necessarily those of highly cost-effective. As such, findings in this study were more informative and convincing to policy marker. Our previous study found that by reallocating part of the budget earmarked for cervical cancer screening to HPV vaccination, China could achieve the goal of cervical cancer elimination by the early 2070s without any increase in total financial outlay [14]. However, if these strategies are implemented under a fixed healthcare budget, this may lead to worsened health inequities because it will result in displacement in healthcare spending that may benefit other people (e.g., reducing the budget originally earmarked for screening in order to support the inclusion of HPV vaccination in NIP, because vaccination is more cost-effective under a fixed budget constraint) [13, 14]. In this study, we synchronously considered outcomes in urban and rural areas to select the best strategy for the entire population with different vaccination characteristics. Hence, the initial financial outlay for the recommended pathway should ideally be obtained through new funds. Lastly, the recommended strategies represented by the optimal pathway in this study change over time as population characteristics and availability of technology advance. For example, we considered strategies as supply of vaccines and screening services increased, and the possibility of switching to the 9-valent vaccine. All of those make the pathway provided by this study more realistic and feasible than an evaluation conducted using static assumptions.

Taking considerations of the price and supply of HPV vaccine and the preference of Chinese authorities, China would likely introduce domestically manufactured HPV vaccines into the NIP [7, 12, 15]. Nonetheless, the current commercial price for domestic vaccines is still too high for the NIP, so the healthcare sector has the responsibility of negotiating a competitive price with manufacturers [17, 19]. Although the production capacity for domestic vaccines is 30 million doses per year according to the manufacturer [55], there is still insufficient vaccine supply to fulfill the peak demand under the optimal strategy at 2024–2025. Therefore, increases in capacity by domestic vaccine manufacturers are needed to support projected demand in the next 3 years [18]. On the screening side, while more than 100 HPV testing technologies have been approved by the National Medical Products Administration for cervical screening in China, many of these lack necessary quality tracking and clinical validation when introduced to the screening program implemented by subnational governments. As such, it is necessary to establish transparent tendering processes for selecting and procurement of HPV tests, and establish corresponding monitoring and management mechanisms to ensure the quality of mass screening services. The national government can negotiate special prices for the HPV detection tests to be used in the screening program, thus making elimination more cost-effective. Furthermore, measures should be taken to improve the adequate availability of effective coverage of cervical cancer treatment modalities, especially in the rural areas [59].

Detailed country-level analyses, taking into account specific local factors important for the effective delivery of vaccination and screening interventions, will be key to maximize the use of scarce resources to accelerate efforts to eliminate cervical cancer, and should be viewed as an important complement to global and regional analyses [3]. The speed of cervical cancer elimination in China not only determines the progress of the Western Pacific Region, but also substantially affects the global strategy of eliminating cervical cancer as a public health problem. Our research illustrates how China can develop an optimal pathway towards cervical cancer elimination. It can also serve as an example for other countries, especially those countries that have similar epidemiological features and access to similar types of HPV-related products as China, such as the WHO prequalified *care*HPV™ test and Cecolin™ vaccine (under WHO prequalification review) [11].

Our study has three major strengths. First, we employed health economic evaluation to assess the costs and benefits of the different strategies for cervical cancer elimination, which makes a strong case for investment by the policymakers in this area. Second, we have developed optimal strategies for a different population from the perspective of health economic evaluation, which makes it possible for China to achieve the goal of cervical cancer elimination while saving net economic costs. This approach highlights the importance of developing the pathway to elimination rather than simply examining the timeframe to achieve elimination. Third, we have fully considered the accessibility of healthcare resources and preferences of policy marking in China when we develop the strategies. As such, the optimal pathway in our study is more likely to be adopted by policy-makers and then applied to the population.

Our study has three major limitations. First, we did not consider the scenario of vaccination for boys and the benefits gained from preventing other HPV infection-related diseases. If the only goal is cervical cancer prevention and elimination, then vaccination for boys is an indirect and less cost-effective means of reaching this goal [60]. We did not incorporate other disease outcomes beyond cervical cancer because our estimates suggest that HPV vaccination integrated into the optimal pathway is already very cost-effective, and population coverage is worth maximizing as soon as possible. Second, the time horizon of our modeling extends to 2100, but future outcomes may be subject to many unforeseeable events that may occur before that. We have assumed a very high coverage for both vaccination and screening. While many countries have achieved 90% or higher coverage for vaccination, achieving high coverage for screening is always a challenge. A further consideration is disruption to health services as a result of emergencies such as the current COVID-2019 pandemic [61, 62]. Our data should be interpreted in the context of the challenges and disruptions that may happen in the health sector from time to time. Third, we did not consider the marginal increase in unit costs as population coverage increases, due to the difficulty in covering hard-to-reach populations. Especially if we use GDP per capita as an alternative threshold to assess the optimal strategies, the marginal costs may be more obvious due to a relatively intensive screening. For example, the strategy of screening every 3 years makes it very difficult and costly to deliver about 100 million screening services per year around 2030 (Additional file 2: Figure S11a).

## Conclusions

China can achieve the goal of cervical cancer elimination by the late 2040s (namely, ahead of the second Chinese Centennial Goals) while saving net economic costs by adopting the optimal pathway of interventions we propose from 2021 (namely, the year of the first Chinese Centennial Goals) onwards. To achieve the goal of elimination, China needs to increase investment for cervical cancer prevention during 2021–2035 and improve investment efficiency. Such investment is worthwhile both from the perspective of health economic evaluation and public health services, because it will eventually save China about $100 billion of costs and avoid 7.5 million cervical cancer cases and 2.5 million cervical cancer deaths in 2021–2100.

## Supplementary Information

**Additional file 1.** CHEERS Checklist.

**Additional file 2: Table S1.** Cervical cancer screening costs. **Table S2.** Model inputs. **Table S3.** Population size in China, 2015. **Table S4.** All-cause mortality in China, 2015. **Table S5.** Cervical cancer incidence and mortality in China, 2015. **Table S6.** Prevalence and type distribution of HPV infection in China. **Table S7.** ICER of strategies that lie on the efficiency frontier compared with next-best strategy: **a)** for 2021–30, **b)** for non-vaccinated cohort, **c)** for bivalent vaccinated cohort and **d)** for 9-valent vaccinated cohort. **Table S8.** ICERs of switch 9-valent vaccination from 2031 onwards compared to continuing bivalent vaccination. **Table S9.** Lifetime costs, effectiveness, and incremental cost-effectiveness for optimal pathway (use GDP per capita as threshold) vs. status quo. **Table S10.** Lifetime costs, effectiveness, and incremental cost-effectiveness for optimal pathway using GDP per capita as threshold vs. optimal pathway using median income as threshold. **Table S11.** Estimated cervical cancer cases and deaths averted for optimal pathway (use GDP per capita as threshold) vs. status quo. **Figure S1.** Model structure. **Figure S2.** Model calibration output: **a)** high-risk HPV prevalence, **b)** HPV type distribution and **c)** cervical cancer incidence and mortality. **Figure S3.** Efficiency frontier of all alternative strategies: **a)** for 2021–30, **b)** for non-vaccinated cohort, **c)** for bivalent vaccinated cohort and **d)** for 9-valent vaccinated cohort. **Figure S4.** Probabilistic sensitivity analyses for optimal pathway vs. status quo: **a)** 0% discount and **b)** 3% discount. **Figure S5.** Deterministic sensitivity analyses for optimal pathway vs. status quo: **a)** in China, **b)** in urban area and **c)** in rural area. **Figure S6.** Annual costs of optimal pathway: **a)** 0% discount and **b)** 3% discount. **Figure S7.** Annual costs of status quo: **a)** 0% discount and **b)** 3% discount. **Figure S8.** Annual incremental benefits of optimal pathway compared to status quo: **a)** 0% discount and **b)** 3% discount. **Figure S9.** Optimal pathway towards cervical cancer elimination in China (use GDP per capita as threshold). **Figure S10.** Age-standardized cervical cancer incidence and mortality by adopting optimal pathway (use GDP per capita as threshold). **Figure S11.** Annual costs of optimal pathway (use GDP per capita as threshold): **a)** 0% discount and **b)** 3% discount. **Figure S12.** Annual incremental benefits of optimal pathway (use GDP per capita as threshold) compared to status quo: **a)** 0% discount and **b)** 3% discount.

## Data Availability

The data generating the findings of this article are included within the article and its additional files.

## Abbreviations

CIs: Confidence intervals
CIN: Cervical intraepithelial neoplasia
GDP: Gross domestic product
HPV: Human papillomavirus
ICER: Incremental cost-effectiveness ratio
LBC: Liquid-based cytology
NIP: National immunization program
QALYs: Quality-adjusted life-years
WHO: World Health Organization

## References

[1] Simms KT, Steinberg J, Caruana M, Smith MA, Lew JB, Soerjomataram I (2019). Impact of scaled up human papillomavirus vaccination and cervical screening and the potential for global elimination of cervical cancer in 181 countries, 2020-99: a modelling study. Lancet Oncol.

[2] Brisson M, Kim JJ, Canfell K, Drolet M, Gingras G, Burger EA (2020). Impact of HPV vaccination and cervical screening on cervical cancer elimination: a comparative modelling analysis in 78 low-income and lower-middle-income countries. Lancet.

[3] Canfell K, Kim JJ, Brisson M, Keane A, Simms KT, Caruana M (2020). Mortality impact of achieving WHO cervical cancer elimination targets: a comparative modelling analysis in 78 low-income and lower-middle-income countries. Lancet.

[4] Li X, Zheng R, Li X, Shan H, Wu Q, Wang Y (2017). Trends of incidence rate and age at diagnosis for cervical cancer in China, from 2000 to 2014. Chin J Cancer Res.

[5] Li S, Hu T, Lv W, Zhou H, Li X, Yang R (2013). Changes in prevalence and clinical characteristics of cervical cancer in the People’s Republic of China: a study of 10,012 cases from a nationwide working group. Oncologist.

[6] ICO/IARC Information Centre on HPV and Cancer (2018). Human papillomavirus and related diseases in China: HPV Information Centre.

[7] Zhao F, Qiao Y (2019). Cervical cancer prevention in China: a key to cancer control. Lancet.

[8] Bao HL, Wang LH, Wang LM, Fang LW, Zhang M, Zhao ZP (2018). Study on the coverage of cervical and breast cancer screening among women aged 35-69 years and related impact of socioeconomic factors in China, 2013. Chin J Epidemiol.

[9] National Medical Products Administration. First domestic recombinant human papillomavirus vaccine approved. https://www.nmpa.gov.cn/yaowen/ypjgyw/20191231160701608.html. Accessed 6 Aug 2020.

[10] Xiamen Innovax. Recombinant human papillomavirus bivalent (types 16, 18) vaccine (*Escherichia coli*). http://www.innovax.cn/research.aspx?BaseInfoCateID=121&CateID=121. Accessed 7 Jul 2020.

[11] Gavi. Gavi-supported HPV vaccines profiles to support country decision making. https://www.gavi.org/sites/default/files/document/2020/Gavi-HPV-vaccine-profiles_May%202020.pdf. Accessed 7 Jul 2020.

[12] National Working Committee on Children and Women under State Council. CPPCC National Committee member Yu Luming: Introducing HPV vaccine into the national immunization program. http://www.nwccw.gov.cn/2020-05/22/content_284491.htm. Accessed 6 Jul 2020.

[13] Malagón T (2019). Reasons for optimism about eliminating cervical cancer in China. Lancet Public Health.

[14] Xia C, Hu S, Xu X, Zhao X, Qiao Y, Broutet N (2019). Projections up to 2100 and a budget optimisation strategy towards cervical cancer elimination in China: a modelling study. Lancet Public Health.

[15] Vaccine and Immunization Branch Chinese Preventive Medicine Association (2019). Expert consensus on immunological prevention of human papillomavirus-related diseases. Chin J Prev Med.

[16] Qiao YL, Wu T, Li RC, Hu YM, Wei LH, Li CG (2020). Efficacy, safety, and immunogenicity of an Escherichia coli-produced bivalent human papillomavirus vaccine: an interim analysis of a randomized clinical trial. J Natl Cancer Inst.

[17] China Global Television Network. China-made HPV vaccine comes to the rescue. https://news.cgtn.com/news/2020-01-09/China-made-HPV-vaccine-comes-to-the-rescue-N7kwyGP9yo/index.html. Accessed 16 Apr 2020.

[18] Gavi. HPV vaccine manufacturers commit to provide enough supply to immunise at least 84 million girls in Gavi countries. https://www.gavi.org/news/media-room/hpv-vaccine-manufacturers-commit-provide-enough-supply-immunise-least-84-million. Accessed 7 Jul 2020.

[19] Yu W, Lu M, Wang H, Rodewald L, Ji S, Ma C (2018). Routine immunization services costs and financing in China, 2015. Vaccine.

[20] Centers for Disease Control and Prevention. CDC vaccine price list. https://www.cdc.gov/vaccines/programs/vfc/awardees/vaccine-management/price-list/. Accessed 16 Apr 2020.

[21] DRUGDATAEXPY. Information of drug winning bid. https://data.yaozh.com/yaopinzhongbiao. Accessed 16 Apr 2020.

[22] Beijing Municipal Health Commission. Optimizing and integrating screening of cancers of cervical and breast in Beijing: Government Notification. http://wjw.beijing.gov.cn/zwgk_20040/fgwj/gfxwj/201912/t20191216_1239755.html. Accessed 21 Apr 2020.

[23] China Hainan Government Procurement. Hainan Women’s and Children’s Medical Center - HPV testing reagents and related services for women’s common diseases and “two cancers” in 2019 - bid winning announcement. https://www.ccgp-hainan.gov.cn/cgw/cgw_show_zbgg.jsp?id=21831. Accessed 24 Apr 2020.

[24] Zhejiang Government Procurement Center. Announcement of bid winning (transaction) results of “two cancer” examination project of provincial health commission, HPV and TCT test reagent project. http://zfcg.czt.zj.gov.cn/innerUsed_noticeDetails/index.html?noticeId=6604290. Accessed 24 Apr 2020.

[25] Jiangsu Public Resource Trading Platform. TCT and HPV test service project of “two cancers” program in Tongzhou District, Nantong City. http://jsggzy.jszwfw.gov.cn/jyxx/003004/003004002/20200421/f67ce97c-e4f6-44b3-977e-4bc17d5930df.html. Accessed 24 Apr 2020.

[26] Peng JR, Tao SY, Wen Y, Yang X, Ma JQ, Zhao F (2019). Cost-effectiveness analysis of cervical cancer screening strategies in urban China. Chin J Oncol.

[27] Zhao FH, Lewkowitz AK, Hu SY, Chen F, Li LY, Zhang QM (2012). Prevalence of human papillomavirus and cervical intraepithelial neoplasia in China: a pooled analysis of 17 population-based studies. Int J Cancer.

[28] Tao SY, Peng JR, Wang Y, Zhang GT, Chen ZY, Zhao F (2018). Study on direct economic burden and influencing factors in patients with cervical cancer and precancerous lesions. Chin J Prev Med.

[29] National Bureau of Statistics of China. Residents’ income and consumption expenditure in 2019. http://www.stats.gov.cn/tjsj/zxfb/202001/t20200117_1723396.html. Accessed 21 Apr 2020.

[30] Haeussler KD. A dynamic Bayesian Markov model for health economic evaluations of interventions in infectious disease. London: UCL (University College London); 2017. 10.1186/s12874-018-0541-7 PMC6090931 30068316

[31] Haeussler K, den Hout AV, Baio G (2018). A dynamic Bayesian Markov model for health economic evaluations of interventions in infectious disease. BMC Med Res Methodol.

[32] Liu YJ, Zhang Q, Hu SY, Zhao FH (2016). Effect of vaccination age on cost-effectiveness of human papillomavirus vaccination against cervical cancer in China. BMC Cancer.

[33] Canfell K, Barnabas R, Patnick J, Beral V (2004). The predicted effect of changes in cervical screening practice in the UK: results from a modelling study. Br J Cancer.

[34] Goldie SJ, Grima D, Kohli M, Wright TC, Weinstein M, Franco E (2003). A comprehensive natural history model of HPV infection and cervical cancer to estimate the clinical impact of a prophylactic HPV-16/18 vaccine. Int J Cancer.

[35] Haeussler K, Marcellusi A, Mennini FS, Favato G, Picardo M, Garganese G (2015). Cost-effectiveness analysis of universal human papillomavirus vaccination using a dynamic Bayesian methodology: the BEST II study. Value Health.

[36] Myers ER, McCrory DC, Nanda K, Bastian L, Matchar DB (2000). Mathematical model for the natural history of human papillomavirus infection and cervical carcinogenesis. Am J Epidemiol.

[37] Yokoyama M, Iwasaka T, Nagata C, Nozawa S, Sekiya S, Hirai Y (2003). Prognostic factors associated with the clinical outcome of cervical intraepithelial neoplasia: a cohort study in Japan. Cancer Lett.

[38] Sawaya GF, Sanstead E, Alarid-Escudero F, Smith-McCune K, Gregorich SE, Silverberg MJ (2019). Estimated quality of life and economic outcomes associated with 12 cervical cancer screening strategies: a cost-effectiveness analysis. JAMA Intern Med.

[39] Johnson HC, Elfstrom KM, Edmunds WJ (2012). Inference of type-specific HPV transmissibility, progression and clearance rates: a mathematical modelling approach. PLoS One.

[40] Abbas KM, van Zandvoort K, Brisson M, Jit M (2020). Effects of updated demography, disability weights, and cervical cancer burden on estimates of human papillomavirus vaccination impact at the global, regional, and national levels: a PRIME modelling study. Lancet Glob Health.

[41] Pan QJ, Hu SY, Zhang X, Ci PW, Zhang WH, Guo HQ (2013). Pooled analysis of the performance of liquid-based cytology in population-based cervical cancer screening studies in China. Cancer Cytopathol.

[42] Ma L, Wang Y, Gao X, Dai Y, Zhang Y, Wang Z (2019). Economic evaluation of cervical cancer screening strategies in urban China. Chin J Cancer Res.

[43] van Rosmalen J, de Kok IM, van Ballegooijen M (2012). Cost-effectiveness of cervical cancer screening: cytology versus human papillomavirus DNA testing. BJOG.

[44] Zhao ZM, Pan XF, Lv SH, Xie Y, Zhang SK, Qiao YL (2014). Quality of life in women with cervical precursor lesions and cancer: a prospective, 6-month, hospital-based study in China. Chin J Cancer.

[45] Haacker M, Hallett TB, Atun R (2020). On discount rates for economic evaluations in global health. Health Policy Plan.

[46] National Bureau of Statistics of China. National data. http://data.stats.gov.cn/english/. Accessed 11 Mar 2020.

[47] National Bureau of Statistics of China (2017). China population and employment statistics yearbook 2016.

[48] National Health and Family Planning Commission (2016). 2016 China health statistics yearbook.

[49] He J (2019). 2018 China Cancer Registry Annual Report.

[50] Bouvard V, Baan R, Straif K, Grosse Y, Secretan B, El Ghissassi F (2009). A review of human carcinogens--part B: biological agents. Lancet Oncol.

[51] National Bureau of Statistics of China. National data. http://data.stats.gov.cn/english/. Accessed 14 Apr 2020.

[52] Choi HCW, Jit M, Leung GM, Tsui KL, Wu JT (2018). Simultaneously characterizing the comparative economics of routine female adolescent nonavalent human papillomavirus (HPV) vaccination and assortativity of sexual mixing in Hong Kong Chinese: a modeling analysis. BMC Med.

[53] Zeng H, Chen W, Zheng R, Zhang S, Ji JS, Zou X (2018). Changing cancer survival in China during 2003-15: a pooled analysis of 17 population-based cancer registries. Lancet Glob Health.

[54] Wang R, Pan W, Jin L, Huang W, Li Y, Wu D (2020). Human papillomavirus vaccine against cervical cancer: opportunity and challenge. Cancer Lett.

[55] China Securities Regulatory Commission. Prospectus of Beijing Wantai Biological Pharmacy Enterprise Co. Ltd. http://www.csrc.gov.cn/pub/zjhpublic/G00306202/201906/P020190606673000620633.pdf. Accessed 7 Jul 2020.

[56] Bao HL (2017). The study of social inequality in cervical cancer mortality and screening for efficient screening pattern in China.

[57] The State Council of People’s Republic of China (2019). The national per capita disposable personal income.

[58] The State Council of People’s Republic of China. China’s GDP totaled 99.09 trillion yuan in 2019. http://www.gov.cn/guoqing/2020-03/09/content_5362699.htm. Accessed 29 May 2020.

[59] GBD 2019 Universal Health Coverage Collaborators (2020). Measuring universal health coverage based on an index of effective coverage of health services in 204 countries and territories, 1990–2019: a systematic analysis for the Global Burden of Disease Study 2019. Lancet.

[60] Arie S (2019). HPV: WHO calls for countries to suspend vaccination of boys. BMJ.

[61] The Lancet Oncology (2020). COVID-19: global consequences for oncology. Lancet Oncol.

[62] Sud A, Torr B, Jones ME, Broggio J, Scott S, Loveday C (2020). Effect of delays in the 2-week-wait cancer referral pathway during the COVID-19 pandemic on cancer survival in the UK: a modelling study. Lancet Oncol.

