# Supplementary material for: Effectiveness and cost-effectiveness of eliminating cervical cancer through a tailored optimal pathway: a modeling study
Source: BMC Med. 2021 Mar 3;19:62. doi: 10.1186/s12916-021-01930-9 (PMC7927373; doi:10.1186/s12916-021-01930-9)
Supplement: Supplementary file 2 — Additional file 2: Table S1. Cervical cancer screening costs. Table S2. Model inputs. Table S3. Population size in China, 2015. Table S4. All-cause mortality in China, 2015. Table S5. Cervical cancer incidence and mortality in China, 2015. Table S6. Prevalence and type distribution of HPV infection in China. Table S7. ICER of strategies that lie on the efficiency frontier compared with next-best strategy: a) for 2021–30, b) for non-vaccinated cohort, c) for bivalent vaccinated cohort and d) for 9-valent vaccinated cohort. Table S8. ICERs of switch 9-valent vaccination from 2031 onwards compared to continuing bivalent vaccination. Table S9. Lifetime costs, effectiveness, and incremental cost-effectiveness for optimal pathway (use GDP per capita as threshold) vs. status quo. Table S10. Lifetime costs, effectiveness, and incremental cost-effectiveness for optimal pathway using GDP per capita as threshold vs. optimal pathway using median income as threshold. Table S11. Estimated cervical cancer cases and deaths averted for optimal pathway (use GDP per capita as threshold) vs. status quo. Figure S1. Model structure. Figure S2. Model calibration output: a) high-risk HPV prevalence, b) HPV type distribution and c) cervical cancer incidence and mortality. Figure S3. Efficiency frontier of all alternative strategies: a) for 2021–30, b) for non-vaccinated cohort, c) for bivalent vaccinated cohort and d) for 9-valent vaccinated cohort. Figure S4. Probabilistic sensitivity analyses for optimal pathway vs. status quo: a) 0% discount and b) 3% discount. Figure S5. Deterministic sensitivity analyses for optimal pathway vs. status quo: a) in China, b) in urban area and c) in rural area. Figure S6. Annual costs of optimal pathway: a) 0% discount and b) 3% discount. Figure S7. Annual costs of status quo: a) 0% discount and b) 3% discount. Figure S8. Annual incremental benefits of optimal pathway compared to status quo: a) 0% discount and b) 3% discount. Figure S9. Optimal pathway tow [file 12916_2021_1930_MOESM2_ESM.pdf]

Effectiveness and cost-effectiveness of eliminating cervical cancer through a tailored optimal pathway: a modelling study

Supplementary appendix

Changfa Xia, Xiaoqian Xu, Xuelian Zhao, Shangying Hu, Youlin Qiao, Yong Zhang, Raymond Hutubessy, Partha Basu, Nathalie Broutet, Mark Jit, Fanghui Zhao

Contents

1. Methods 2

1.1 Model structure 2

1.2 Parameters 3

1.3 Demographic and epidemiological data 9

1.4 Model calibration 12

1.5 Strategy selection 16

2. Results 26

2.1 Sensitivity analysis 26

2.2 Annual costs and benefits 32

2.3 Use GDP per capita as threshold 39

2.3.1 Main results 39

2.3.2 Annual costs and benefits 44

3. References 49

1. Methods

1.1 Model structure

Model structure shown in Figure S1 illustrates the sexual mixing between males and females, and the natural history of high-risk human papillomavirus (HPV) infection and cervical cancer among females. This is a hybrid model consisting a dynamic model and a natural history model. We used the dynamic model to simulate the HPV transmission between males and females, whereas the natural history model was employed to simulate the natural history of cervical cancer and to obtain the number of cervical cancer cases and deaths associated with HPV infections.

Sexual mixing matrices for every stratum were calculated by use of partner acquisition rates and assortativity of age, area, and sexual activity [1]. Partner acquisition rates were adjusted to maintain the number of male-female sexual partnerships [1]. Neonates are assumed to be in the uninfected state at birth, and all-cause mortality was present in all states. Each individual was entered into the model at their age in 2015 or the year of their birth, if after 2015 and, within the model, they were randomly allocated to a new state, based on the transition probabilities. Individuals were transitioned between states representing no current or previous infection or vaccine (in which they were considered susceptible to infections); immunity (including infection-acquired immunity and vaccine-acquired immunity); infection; development of cervical intraepithelial neoplasia (CIN) grade 1, CIN2, or CIN3 disease (representing mild, moderate, and severe dysplasia); effective detection and treatment; and cervical cancer likely to cause death or to extend until the simulation end point [1]. We assumed that infection-acquired immunity will wane over time, whereas vaccine-acquired immunity will be lifelong. If HPV vaccination is available, individuals aged 9 years were considered to be vaccinated at predefined coverages in the dynamic model. Cervical cancer screening fits in the natural history model where screening is available [1].

Local cervical cancer without symptoms may become symptomatic or progress to more advanced stages of cervical cancer without symptoms. In the absence of screening, cervical cancer is diagnosed only when symptoms develop in which the patient is immediately treated. Females with symptomatic cervical cancer are subjected to the age-specific death and cure probability of cervical cancer. Cancer patients who remain alive 5 years after cancer diagnosis are moved to the health state “cured” and are break from the model in the rest of simulations.

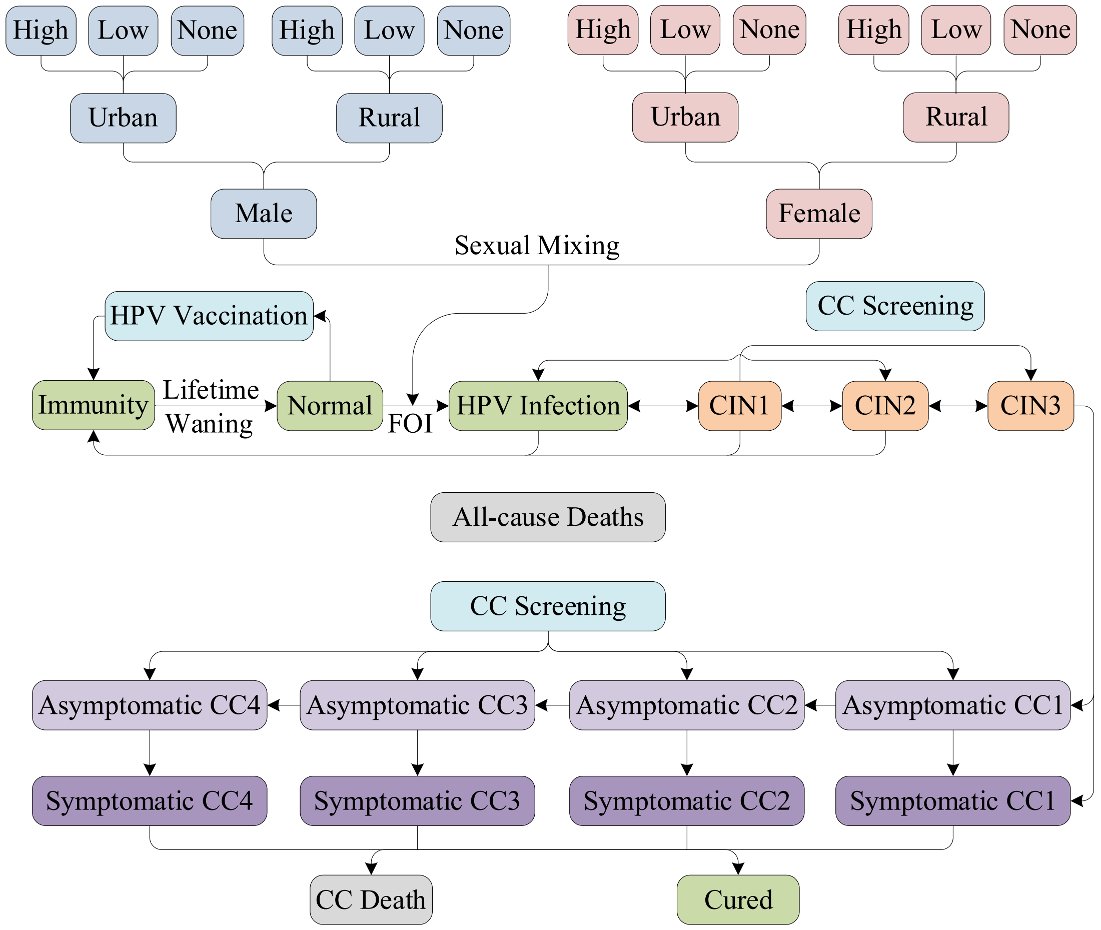

Figure S1. Model structure

Individuals with multiple sexual partners are regarded as having high sexual activity level, while individuals with only one sexual partner are regarded as having low sexual activity level. Abbreviations: HPV, human papillomavirus; FOI, force of infection; CIN, cervical intraepithelial neoplasia; CC, cervical cancer.

1.2 Parameters

Taking considerations of the price of HPV vaccine and the preference of Chinese authorities, China would most likely to introduce domestically-manufactured HPV vaccine into its national immunization program (NIP) [2-4]. The first Chinese domestic *Escherichia coli*-produced bivalent human papillomavirus vaccine (Xiamen Innovax) has been approved by the National Medical Products Administration (NMPA) in 2019 [5, 6], and its first commercial price (about $46 per dose) is much lower than that of imported bivalent vaccine [7]. In June 2020, another domestic HPV vaccine manufacturer announced that Walvax expects to get approved by Chinese regulatory authorities in 2021, followed by an application to WHO for prequalification [8]. By July 2020, there are more than 10 domestically-manufactured vaccines are undergoing phase II or phase III clinical

trials in China. There is a possibility of price competition between HPV vaccine manufacturers in a few years. Moreover, the price of the domestically-manufactured HPV vaccine is likely to be as low as $3.00 per dose [8, 9]. It is noteworthy that the total price of 22 doses of Type 1 vaccines included in the National Expanded Program on Immunization (EPI) in China was only $19.08/child in 2015 [10]. As such, we assumed the cost of the government-delivered bivalent HPV vaccine would be the same as that enabled by Gavi, the Vaccine Alliance, namely $4.6 per dose for Cervarix [9].

The price of government-delivered 9-valent HPV vaccine was estimated by two approaches of mutual corroboration: 1). Prices of US CDC-delivered 9-valent HPV vaccine (Merck) and Hepatitis B vaccine (Merck) are $187.01 and $12.53 per dose [11], while the price of Hepatitis B vaccine from Chinese CDC is only $0.43 (3.04 Chinese yuan [CNY]) per dose [12]. Using the price ratio from US, we estimated the price of 9-valent HPV vaccine would be $6.48 per dose. 2). Prices of government-delivered and commercial Hepatitis B vaccine (both 10 micrograms of yeast-derived recombinant hepatitis B vaccine) in China are $0.43 (3.04 CNY) and $11.93 (83.5 CNY) per dose, while the commercial price of 9-valent HPV vaccine is $185.43 (1298 CNY) per dose [12]. Using the price ratio of commercial and government-delivered vaccines, we estimated the price of 9-valent HPV vaccine would be $6.75 per dose. Combining the estimates from the above two approaches, the price of Chinese government-delivered 9-valent HPV vaccine would be $6.6 per dose in 2031 onwards.

Screening strategy using HPV DNA testing as primary screening (with genotyping) and cytology triage was simulated in our model, namely women positive by HPV16/18 will be referred to colposcopy and women positive by other 11 oncogenic types will be triaged with liquid-based cytology (LBC). We adopt the prices and proportions provided by cost calculation table of cervical cancer screening program organized by the government. However, the prices of HPV-test vary greatly in different cities and counties. For example, price of HPV-test in Beijing is estimated at 90 CNY [13], while the unit purchase price of HPV-test reported in Hainan province is only 19 CNY [14]. Considering that the price of HPV-test in China is constantly decreasing and the unit price of government procurement in many regions is below 40 CNY [14-16], the materials price of HPV-test would be the same of *careHPV* test, namely 35 CNY. HPV-testing related equipment costs, personnel costs, non-medical costs, and indirect cost are estimated based on our previous national-wide cervical cancer screening demonstration research project and the prices have been converted to that in 2020 [17]. Consistent with government provided cost calculation table and our population-based pooled data, we estimate that 15% of women will receive a positive results of high-risk HPV infection with HPV-testing as primary screening test, and 4% of women will be positive for HPV16/18 infection [18, 19].

Table S1. Cervical cancer screening costs (Chinese yuan, CNY)

**Subjects**		**Urban area**		**Rural area**	
		**Unit price**	**Subtotal**	**Unit price**	**Subtotal**
Gynecological examination		15	15	15	15
HPV test	Materials	35	35	35	35
	Equipment[17]	0.5	0.5	0.5	0.5
	Personnel costs[17, 20]	15	15	6	6
	Non-medical costs[17]	5	5	2	2
	Indirect cost[17]	Direct cost ×25%	13.88	Direct cost ×25%	10.88
LBC (11% from HPV test)		66	7.26	66	7.26
Colposcopy (50% from LBC and 4% from HPV test)		60	5.7	60	5.7
Medical service fee of colposcopy referral		10	0.95	10	0.95
Histopathology (50% from colposcopy)		160	7.6	160	7.6
**Total**	CNY (Chinese yuan)	..	105.89	..	90.89
	USD (United States dollar)	..	15.13	..	12.98

Abbreviations: HPV, human papillomavirus; LBC, liquid-based cytology.

Treatment costs of CIN and cervical cancer (CC) include direct medical cost, direct non-medical costs and indirect costs. Data of direct costs and the ratio of indirect costs and direct costs are extracted from our previous national-wide cervical cancer screening demonstration research project and the prices have been converted to that in 2020 [17, 21]. Although the cervical cancer screening guidelines recommend follow-up rather than treatment for CIN1, about 45% of Chinese women diagnosed with CIN1 will seek to medical treatment. The costs of CIN1 treatment are estimated by consultations of clinical experts providing CIN1 treatment services [1].

For newly diagnosed disease, the quality-adjusted life-years (QALYs) lost were calculated by aggregating the quality-of-life loss from pre-treatment, 1-month posttreatment, and 3-months posttreatment of precancer and cancer. For previously diagnosed diseases, we calculated QALYs using the quality of life of patients 6 months post-treatment after excluding individuals receiving 1-month terminal care and eventually dying from cervical cancer.

The range of values tested in the sensitivity analysis for each parameter was either based on the

reported 95% confidence intervals in the referenced studies or determined by assuming a 25% change from the base-case value (Table S2).

Table S2. Model inputs

Parameter	Base case	Range	Distribution	Source
**Transition probabilities**				
HPV clearance				
12–24 years	0.7188	0.6463–0.7830	β	[22-26]
25–29 years	0.6984	0.5898–0.7952	β	[22-26]
30–39 years	0.3503	0.2860–0.4188	β	[22-26]
40–49 years	0.2048	0.1118–0.3022	β	[22-27]
>50 years	0.1004	0.0546–0.1567	β	[22-26]
Infection to CIN1	0.0717 (0.2/36 months)	0.0527–0.1121 (0.15–0.30/36 months)	β	[25, 28]
CIN1 to CIN2	0.2240	0.1608–0.2972	β	[22-26]
CIN2 to CIN3	0.3498	0.2623–0.4372	β	[22-26]
CIN3 to CCI	0.1019	0.0764–0.1274	β	[22-26]
CIN1 to infection/immunity	0.7008	0.6077–0.7933	β	[22-26]
CIN2 to CIN1	0.2494	0.1994–0.2992	β	[22-26]
CIN3 to CIN2	0.0135	±25%	β	[25]
Infection to CIN2	0.0115	0.0034–0.0234	β	[22-26]
CIN2 to infection/immunity	0.1901	±25%	β	[25, 29]
CIN1 to CIN3	0.0464	0.0098–0.1297	β	[22-26]
aCCI to aCC II	0.4377 (0.9/4 years)	±25%	β	[28, 30]
aCCII to aCC III	0.5358 (0.9/3 years)	±25%	β	[28, 30]
aCC III to aCC IV	0.6838 (0.9/2 years)	±25%	β	[28, 30]
Waning of natural immunity	0.021	0.015–0.027	β	[31]
**Cancer symptoms**				
CC FIGO I	0.15	±25%	β	[28, 30]
CC FIGO II	0.225	±25%	β	[28, 30]

CC FIGO III	0.6	±25%	β	[28, 30]
CC FIGO IV	0.9	±25%	β	[28, 30]
**Vaccine efficacy**				
Vaccine types	100%	80–100%	Binomial	Assumed[32-34]
Cross-protect (bivalent)	0%	0–50%	Binomial	Assumed
**Screening sensitivity**				
Liquid-based cytology				
CIN1	0.70	±25%	β	[35]
CIN2/3	0.810	0.782–0.835	β	[35]
CC FIGO I	0.90	±25%	β	Assumed
CC FIGO II	0.92	±25%	β	Assumed
CC FIGO III	0.95	±25%	β	Assumed
CC FIGO IV	0.99	±25%	β	Assumed
HPV test				
CIN1	0.80	±25%	β	Assumed
CIN2	0.90	±25%	β	Assumed[33]
CIN3	0.94	±25%	β	Assumed[33]
CC	1.00	..	..	Assumed
**Precancer management**				
Urban area				
Follow-up of CIN1	0.4631	0.4168–0.5094	β	[1]
Directly treatment of CIN1	0.4421	0.3979–0.4863	β	[1]
Treatment of CIN2/3	0.9533	0.8580–1.0000	β	[1]
Rural area				
Follow-up of CIN1	0.3871	0.3484–0.4258	β	[1]
Directly treatment of CIN1	0.4535	0.4082–0.4989	β	[1]
Treatment of CIN2/3	0.8946	0.8051–0.9841	β	[1]
**Treatment efficacy**				
Urban area				
CIN1	1.0000	0.9956–1.0000	..	[1]
CIN2/3	0.9367	0.9278–0.9455	β	[1]

Rural area				
CIN1	0.9978	0.9943–1.0000	β	[1]
CIN2/3	0.9000	0.8846–0.9154	β	[1]
**Costs (2020 US$)**				
Bivalent vaccine price	4.6	±25%	γ	See context
9-valent vaccine price	6.6	±25%	γ	See context
Vaccination service cost	3.83	±25%	γ	[10]
Urban area				
LBC-based screening	9.64	±25%	γ	[1]
HPV-based screening	15.13	±25%	γ	See context
CIN1 treatment	250.93	170.16–280.82	γ	Delphi method[1]
CIN2 treatment	1817.77	1665.99–1969.55	γ	[17, 21, 36]
CIN3 treatment	2000.16	1859.88–2140.44	γ	[17, 21, 36]
CC FIGO I–IIa treatment	7974.19	7403.87–8544.52	γ	[17, 21, 36]
CC FIGO IIb–IV treatment	14051.52	12557.9–15545.14	γ	[17, 21, 36]
Rural area				
LBC-based screening	7.00	±25%	γ	[1]
HPV-based screening	12.98	±25%	γ	See context
CIN1 treatment	177.92	143.41–245.08	γ	Delphi method[1]
CIN2 treatment	881.99	785.05–978.93	γ	[17, 21, 36]
CIN3 treatment	1314.45	1171.98–1456.93	γ	[17, 21, 36]
CC FIGO I–IIa treatment	5329.05	4363.42–6294.69	γ	[17, 21, 36]
CC FIGO IIb–IV treatment	8819.70	6606.37–11033.02	γ	[17, 21, 36]
**Utilities (quality of life)**				
CIN1 (last for 6 months)	0.97	±25%	β	[37]
CIN2/3 (last for 1 year)				
Pretreatment	0.90	0.8734–0.9266	β	[38]
1 month posttreatment	0.96	0.9445–0.9755	β	[38]
3 months posttreatment	0.98	0.9445–0.9944	β	[38]
6 months posttreatment	0.99	0.9856–0.9944	β	[38]
CC FIGO I–IIa				

Pretreatment	0.83	0.7875–0.8725	β	[38]
1 month posttreatment	0.77	0.7232–0.8168	β	[38]
3 months posttreatment	0.84	0.7975–0.8825	β	[38]
6 months posttreatment	0.94	0.9145–0.9655	β	[38]
CC FIGO IIb–IV				
Pretreatment	0.84	0.7731–0.9069	β	[38]
1 month posttreatment	0.68	0.5674–0.7926	β	[38]
3 months posttreatment	0.75	0.6409–0.8591	β	[38]
6 months posttreatment	0.86	0.8213–0.8987	β	[38]
Terminal care (last for 1 month)	0.288	±25%	β	[37]
Discount rate	3%	0–5%	..	[39]

Abbreviations: HPV, human papillomavirus; CIN, cervical intraepithelial neoplasia; CC, cervical cancer; aCC, asymptomatic cervical cancer; FIGO, International Federation of Gynecology and Obstetrics; US$, United States dollar.

1.3 Demographic and epidemiological data

We extracted demographic and epidemiological data from open-source publications or government-released online datasets. Population size in 2015 was obtained from National Bureau of Statistics of China (Table S3) [40, 41]. All-cause mortality data was obtained from China Health Statistics Yearbook released by National Health Commission of China (Table S4) [42]. Cervical cancer incidence and mortality data was obtained from Chinese Cancer Registry Report released by National Cancer Center of China (Table S5) [43]. HPV type distributions in women with normal cervical cytology, low grade cervical precancerous lesions, high grade cervical precancerous lesions, and invasive cervical cancer were obtained from the HPV Information Centre (Table S6) [19]. The data sources and analytical methods of HPV prevalence, fertility rate, sexual activity, and screening coverage were reported in our previous study [1].

Table S3. Population size in China, 2015

**Age**	**Urban area**		**Rural area**	
	**Male**	**Female**	**Male**	**Female**
0–4	24661119	21473062	19907493	16878631
5–9	18297966	15499146	22809169	19025832

10–14	18240048	15352288	20298708	17151929
15–19	20665163	17819613	19643732	16899875
20–24	32528982	30446932	19536481	17545159
25–29	42135977	40344460	22665696	23055797
30–34	32175622	31645970	18920693	18487365
35–39	31187281	30157139	18298009	17374265
40–44	36211138	34455942	23775068	23006225
45–49	35532960	33793282	27282109	27042701
50–54	29362884	27920003	23307974	23413895
55–59	20848304	20193448	18207000	17565336
60–64	19339854	19845980	19598163	19200916
65–69	13173507	13593211	14186395	13795226
70–74	8542608	9146779	9245137	9315753
75–79	6310468	7016699	6279698	6866380
80–84	3745346	4502950	3445810	4609174
85+	2053949	2939919	1719489	3098719
**Total**	395013177	376146823	309126823	294333177

Table S4. All-cause mortality (deaths per 100 000 persons) in China, 2015

**Age**	**Urban area**		**Rural area**	
	**Male**	**Female**	**Male**	**Female**
0	482.74	391.82	427.29	339.09
1–4	41.99	34.28	50.73	39.08
5–9	20.42	15.09	25.59	16.08
10–14	24.79	16.55	33.55	18.16
15–19	32.40	13.85	51.60	21.43
20–24	34.06	13.82	50.62	19.57
25–29	62.23	27.28	98.69	38.15
30–34	80.05	37.63	132.70	52.08
35–39	100.74	42.32	152.27	59.62
40–44	196.82	82.14	242.09	95.78
45–49	256.45	111.89	342.22	140.30

50–54	599.41	258.35	643.88	311.47
55–59	671.54	279.36	729.29	336.29
60–64	1296.56	624.67	1347.40	693.03
65–69	2126.89	1123.5	2388.84	1360.54
70–74	3172.67	1871.25	3670.80	2340.54
75–79	5069.34	3401.48	5781.57	3852.60
80–84	9513.94	7138.94	9483.74	6941.25
85+	20754.73	17406.88	19080.16	16185.8
**Total**	711.79	529.40	759.88	562.48

Table S5. Cervical cancer incidence and mortality (per 100 000 women) in China, 2015

**Age**	**Incidence**		**Mortality**	
	**Urban area**	**Rural area**	**Urban area**	**Rural area**
0	0.16*	0	0	0
1–4	0	0	0	0
5–9	0	0	0	0
10–14	0	0	0	0
15–19	0.05	0.07	0	0
20–24	0.34	0.47	0.02	0.13
25–29	2.77	2.99	0.39	0.62
30–34	7.10	6.56	0.92	1.17
35–39	13.75	14.08	2.09	2.13
40–44	21.78	24.15	4.28	4.25
45–49	31.64	35.41	7.67	7.27
50–54	37.49	38.61	10.14	10.06
55–59	31.24	32.27	9.34	9.78
60–64	26.82	32.03	8.79	10.63
65–69	23.75	30.13	9.98	14.36
70–74	20.62	25.80	11.08	14.72
75–79	17.23	23.85	12.81	18.16
80–84	13.84	20.42	17.16	20.26
85+	10.23	12.92	17.05	14.93
**Total**	15.75	16.73	4.94	5.21

*The value in the model simulation is assigned to 0.

Table S6. Prevalence (%) and type distribution of HPV infection in China

Type	Vaccine	Normal cytology	LSIL	HSIL	Invasive cancer
16	Bivalent/9-valent	2.6 (2.5–2.8)	15.9 (14.8–17.1)	37.1 (35.8–38.3)	59.5 (58.3–60.7)
18	Bivalent/9-valent	1.1 (1.0–1.2)	6.4 (5.6–7.2)	7.0 (6.4–7.6)	9.6 (8.9–10.4)
31	9-valent	0.8 (0.7–0.9)	3.5 (3.0–4.2)	5.2 (4.7–5.8)	2.8 (2.4–3.2)
33	9-valent	1.1 (1.0–1.2)	6.2 (5.4–7.0)	7.0 (6.4–7.6)	3.5 (3.0–4.0)
35	..	0.2 (0.2–0.3)	0.8 (0.5–1.1)	0.5 (0.3–0.7)	0.6 (0.4–0.9)
39	..	0.6 (0.6–0.7)	4.9 (4.2–5.7)	2.6 (2.2–3.1)	1.5 (1.2–1.9)
45	9-valent	0.4 (0.3–0.4)	0.9 (0.7–1.3)	1.7 (1.4–2.1)	1.9 (1.4–2.4)
51	..	0.5 (0.4–0.5)	5.2 (4.6–6.0)	3.9 (3.4–4.4)	0.4 (0.2–0.8)
52	9-valent	2.7 (2.6–2.8)	16 (14.8–17.2)	17.6 (16.6–18.6)	6.5 (5.9–7.1)
56	..	0.4 (0.4–0.5)	4.7 (4.0–5.4)	2.1 (1.8–2.6)	1.1 (0.8–1.5)
58	9-valent	1.7 (1.6–1.8)	12.6 (11.6–13.8)	15.7 (14.8–16.6)	8.2 (7.6–8.9)
59	..	0.4 (0.3–0.4)	3.1 (2.6–3.7)	2.7 (2.3–3.1)	2.6 (2.2–3.1)
68	..	0.9 (0.9–1.0)	5.3 (4.6–6.1)	2.8 (2.4–3.3)	1.0 (0.7–1.4)

Abbreviations: LSIL, low-grade intraepithelial lesion; HSIL, high-grade intraepithelial lesion.

1.4 Model calibration

We calibrated the models using a three-stage process by referring epidemiological data of HPV prevalence, cervical incidence and mortality in 2015, HPV type distributions in women with normal cervical cytology, low grade cervical precancerous lesions, high grade cervical precancerous lesions, and invasive cervical cancer. All calibration process was conducted in all population adhering to the status quo status strategy and the assumption that demographic characteristic remained unchanged. Firstly, estimated annual HPV prevalence from dynamic model was calibrated to that from population-based epidemiological study. Secondly, results from calibrated dynamic model were fed into the natural history model to estimate the HPV type-specific cervical cancer incidence. Incidence and HPV type distributions were calibrated by rerunning the model until the mean squared error does not decrease any more. Lastly, estimated cervical cancer mortality was calibrated to data from Chinese Cancer Registry. Model calibration output are shown in the following figures (Figure S2).

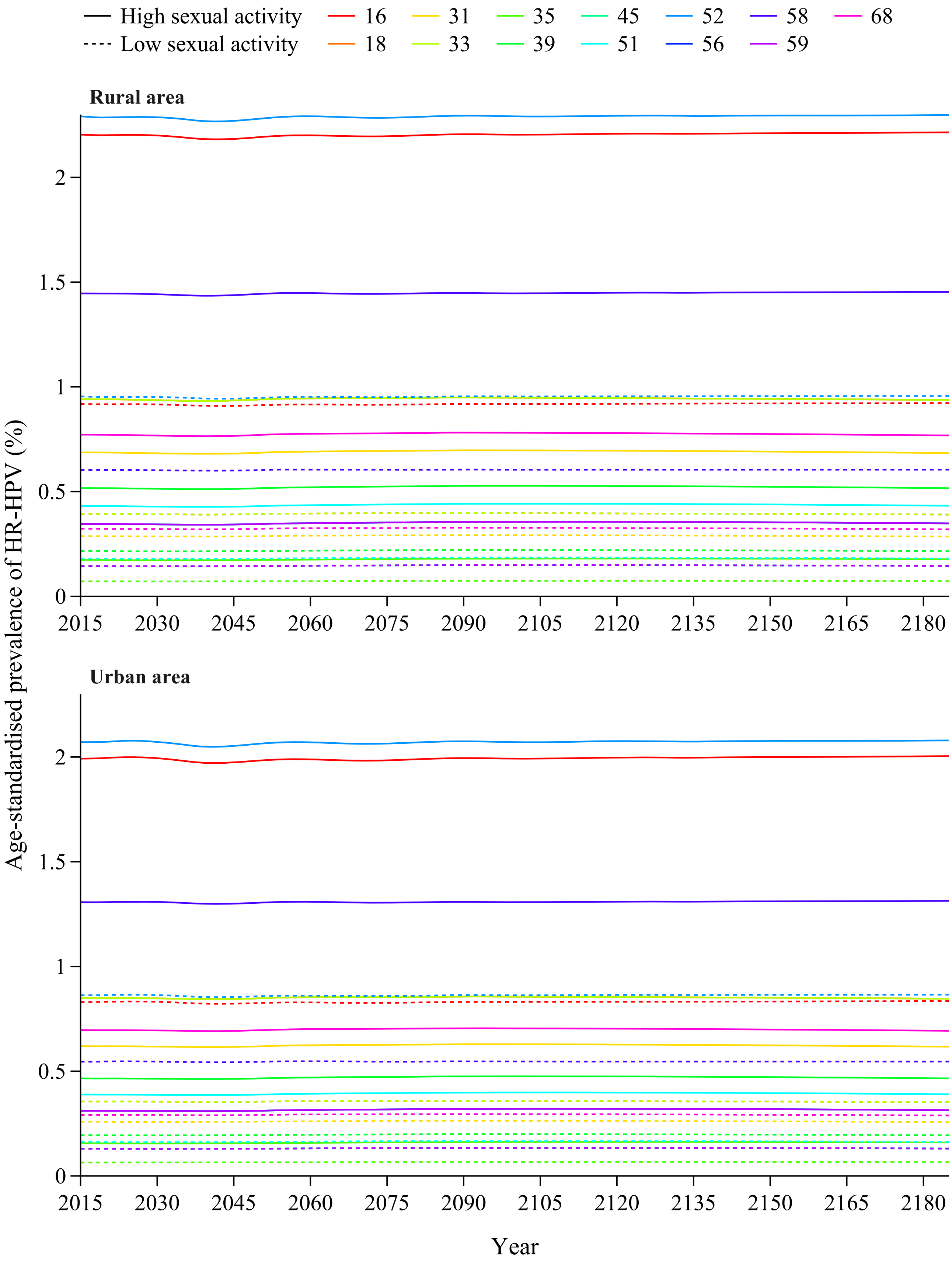

Figure S2a. Model calibration output: high-risk HPV prevalence

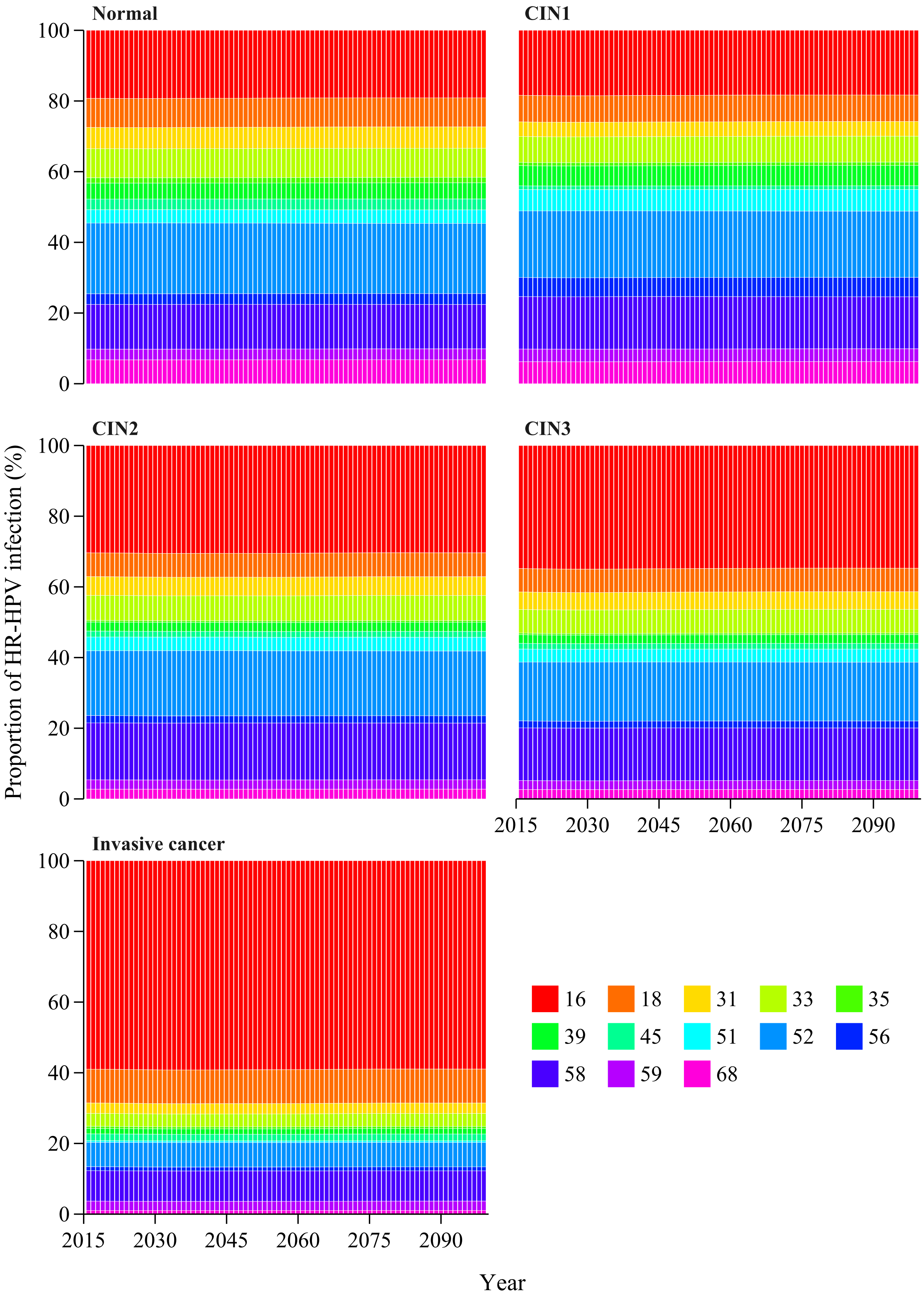

Figure S2b. Model calibration output: HPV type distribution

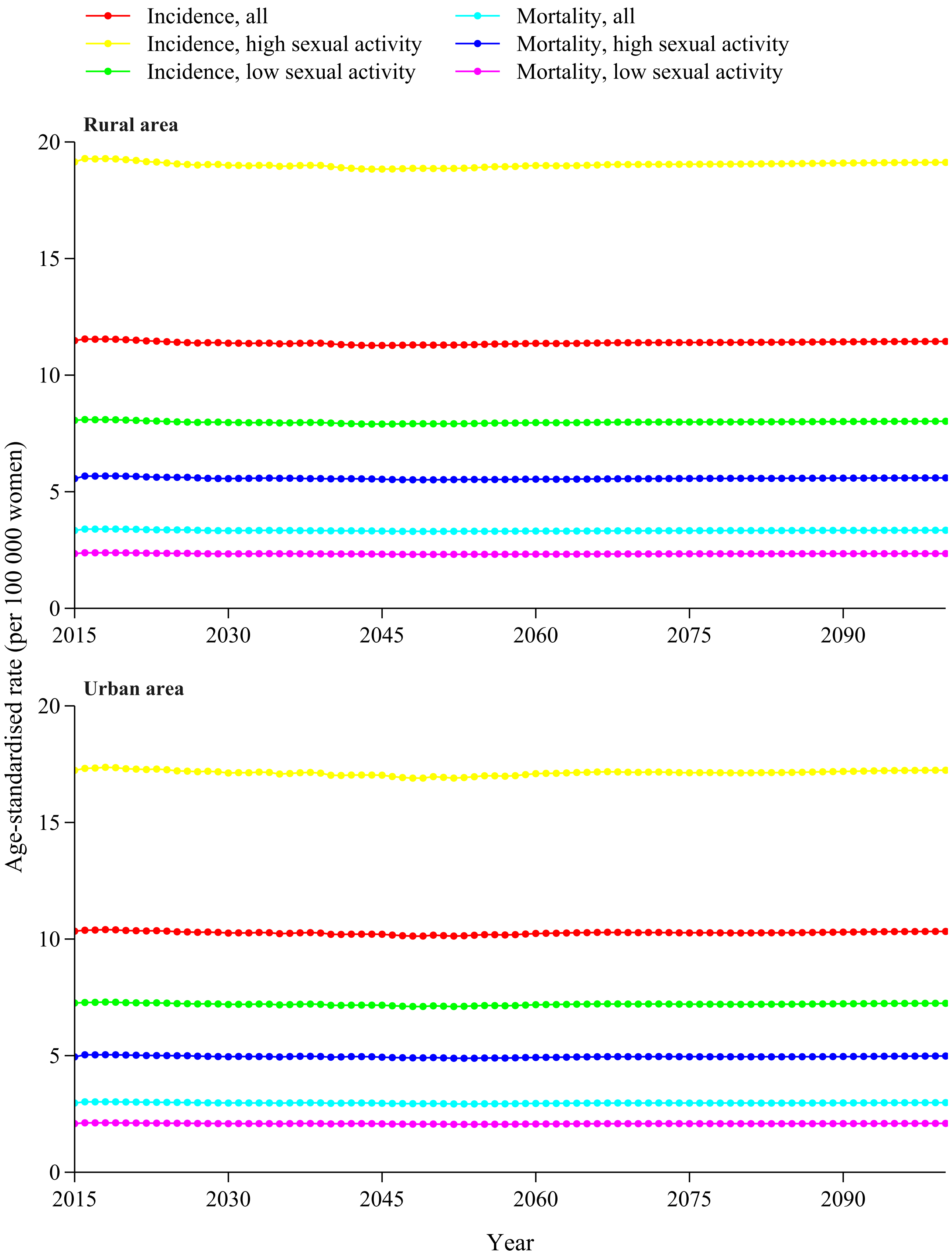

Figure S2c. Model calibration output: cervical cancer incidence and mortality

1.5 Strategy selection

A five-stage analytic process was involved to determine the optimal pathway. Firstly, we evaluated all alternative strategies compared to no intervention in 2021–30 to determine the optimal strategy around HPV vaccination and catch-up vaccination. Secondly, screening strategies for unvaccinated birth cohorts were evaluated and the optimal strategy was then selected. Thirdly and fourthly, we determined the optimal screening strategy for bivalent and 9-valent vaccinated birth cohorts, respectively. Finally, a post-hoc analysis was employed to test whether it was worthwhile to switch from bivalent to 9-valent vaccine in 2031.

The results of five-stage analytic process designed for the selection of optimal pathway were presented at efficiency frontier and incremental cost-effectiveness ratio (ICER) of strategies that lie on the efficiency frontier. Using median income as the ICER threshold, the optimal strategy for routine HPV vaccination, catch-up vaccination, and screening for different birth cohorts were the dominant strategies from a cost-effectiveness perspective.

In stage one analysis in 2021–30, we found that strategy of routine vaccination for 95% of girls aged 9 and catch-up vaccination for girls aged 10–25 is the essential components of the optimal pathway (Figure S3a and Table S7a). However, the optimal screening strategy in 2021–30 (all women are unvaccinated cohorts) either every 3 years or every 5 years need to be determined in stage two analysis for unvaccinated cohorts. Results from stage two analysis for unvaccinated cohorts suggested that screening at 35–64, every 5 year was the optimal strategy when using median income as threshold, while screening at 35–64, every 3 year was the optimal strategy if we use gross domestic product (GDP) per capita as threshold (Figure S3b and Table S7b). In stage three analysis for bivalent vaccinated cohorts, we found that the optimal screening strategy was that screening at 40–55, every 5 years if using median income as threshold, and screening at 35–64, every 5 years if using GDP per capita as threshold (Figure S3c and Table S7c). In stage four analysis for 9-valent vaccinated cohorts, we found that the optimal screening strategy was that screening at 40, 45, and 55 if using median income as threshold, and screening at 40–55, every 5 years if using GDP per capita as threshold (Figure S3d and Table S7d). In stage five post-hoc analysis, discounted ICER for 9-valent vaccine was $-4077/QALY compared to bivalent vaccine (Table S8).

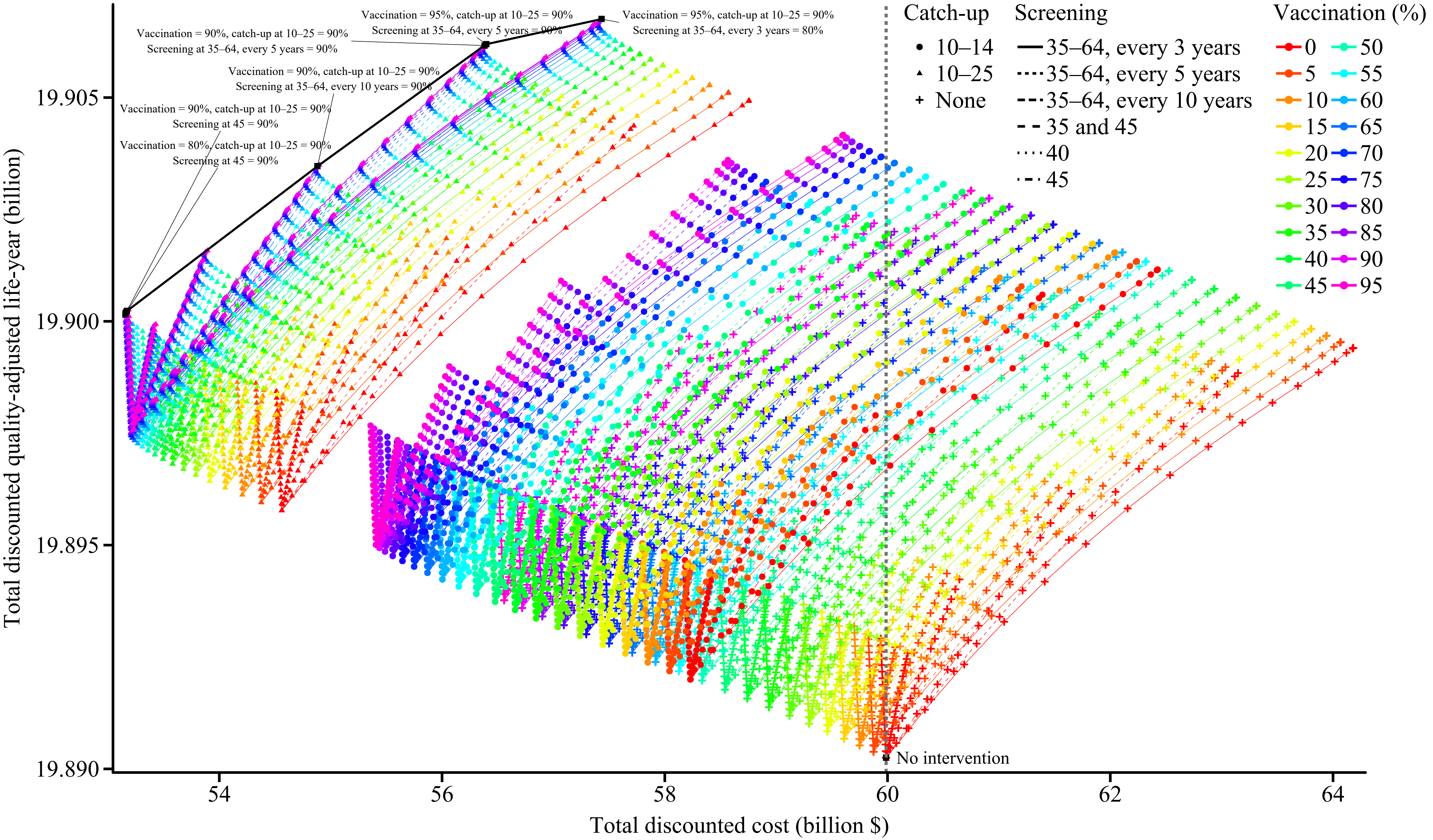

Figure S3a. Efficiency frontier of all alternative strategies in 2021–30

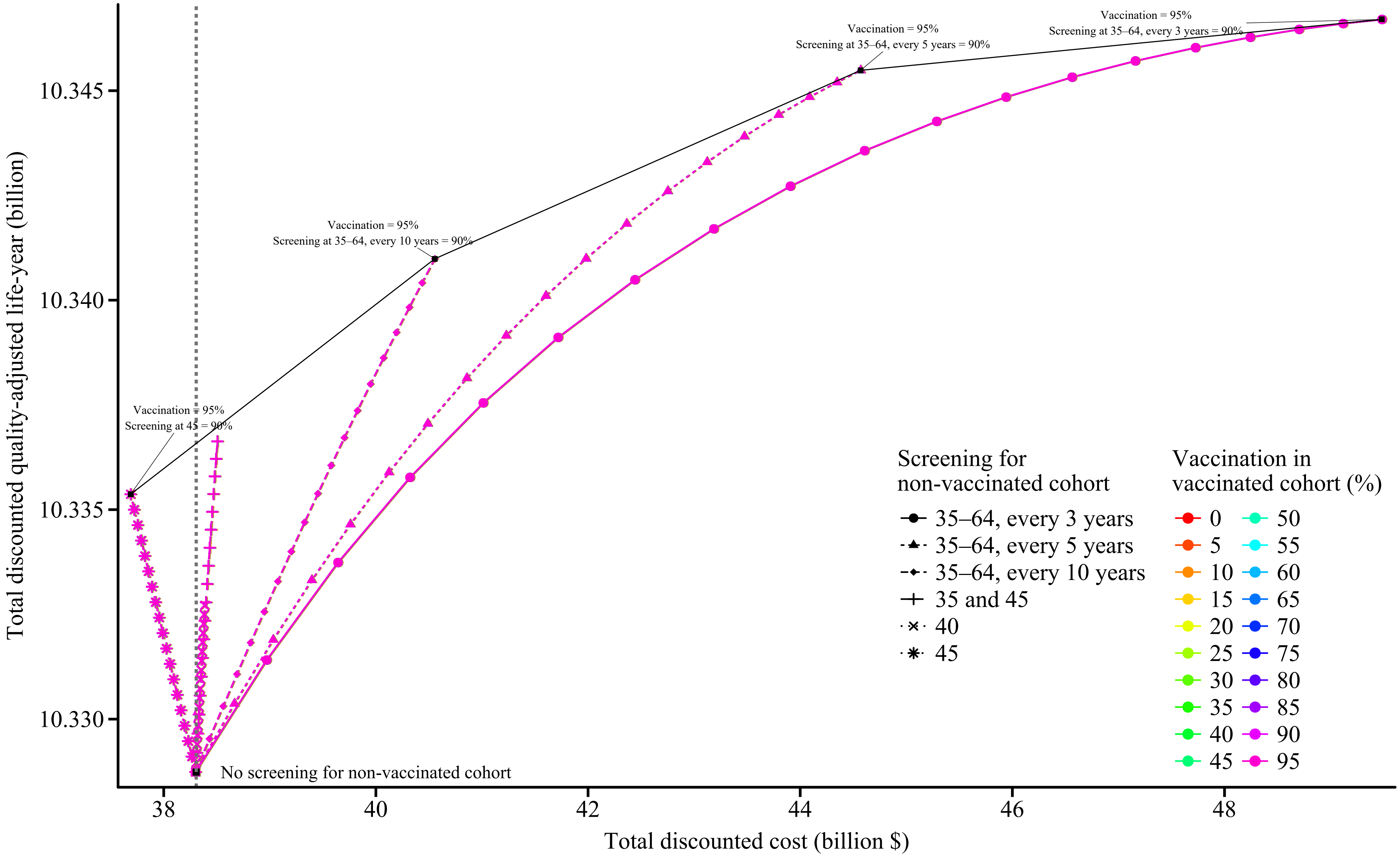

Figure S3b. Efficiency frontier of all alternative strategies for non-vaccinated cohort

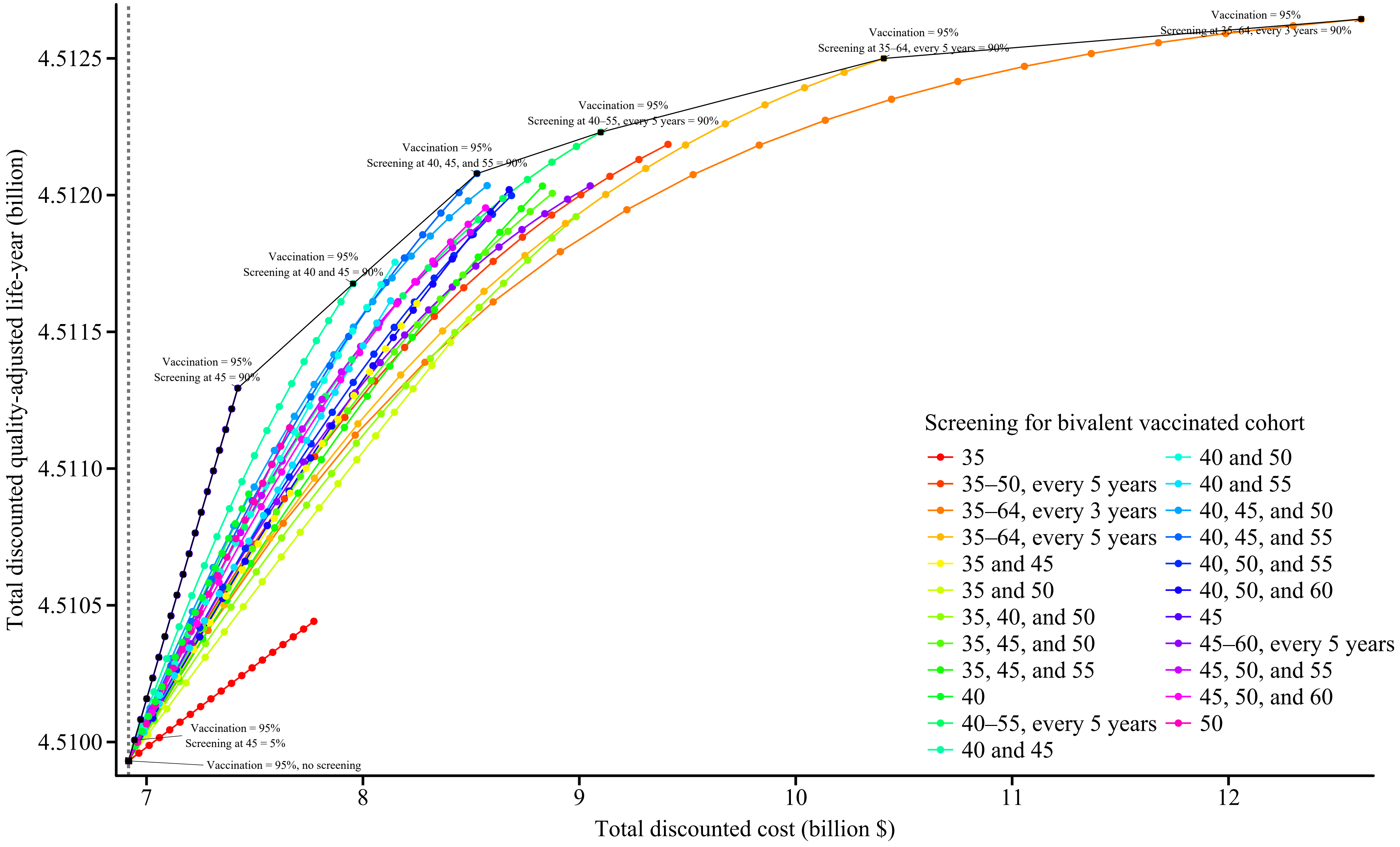

Figure S3c. Efficiency frontier of all alternative strategies for bivalent vaccinated cohort

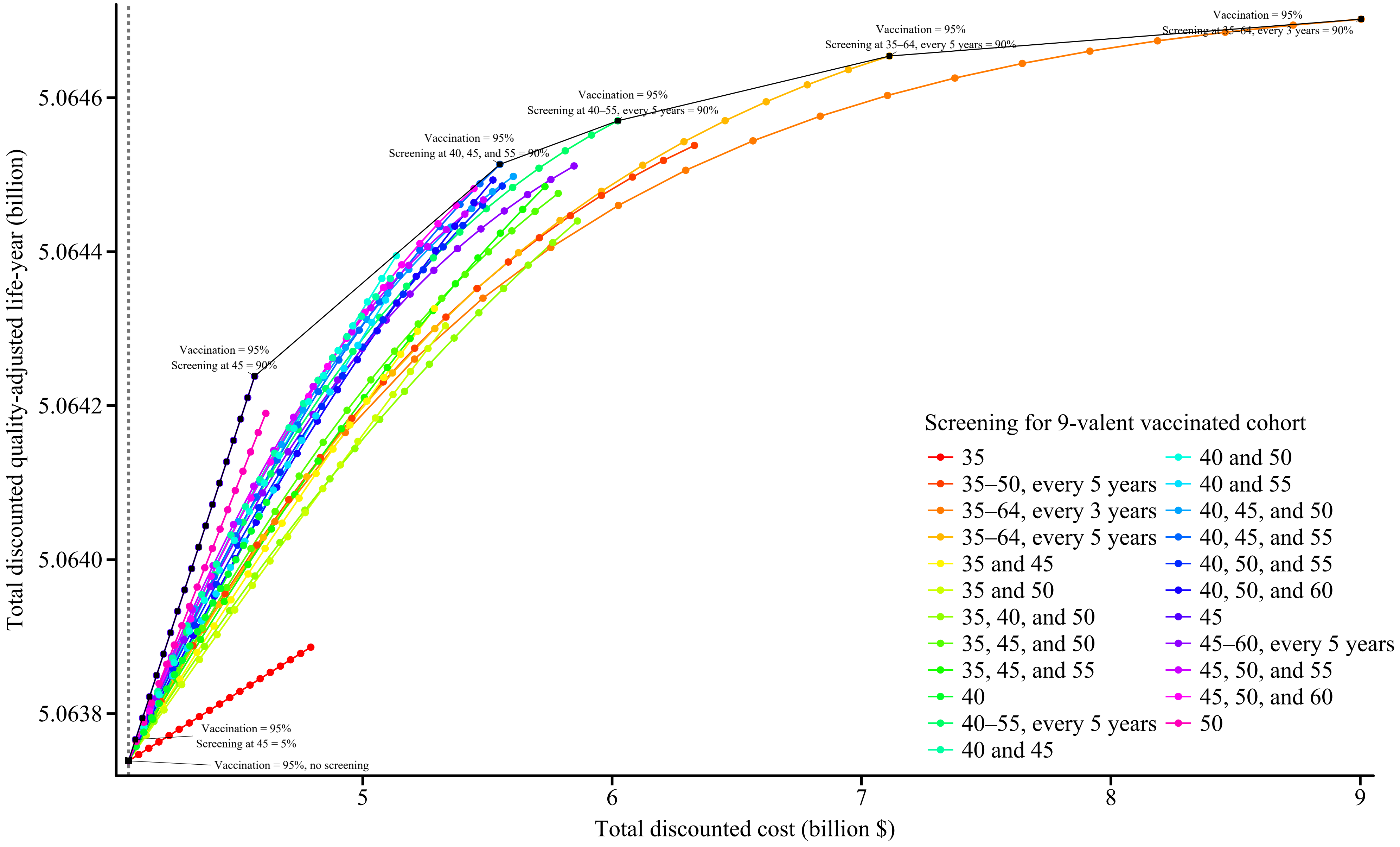

Figure S3d. Efficiency frontier of all alternative strategies for 9-valent vaccinated cohort

Table S7a. ICER of strategies that lie on the efficiency frontier compared with next-best strategy in 2021–30

Vaccination coverage (%)	Catch−up age (90%)	Screening	Screening coverage (%)	Discounted costs	Discounted QALY	Incremental costs	Incremental QALY	ICER
80	10–25	45	90	53155718108	19900157325	..	..	..
85	10–25	45	90	53159978573	19900196886	4260465	39562	108
90	10–25	45	90	53170937661	19900231154	10959087	34267	320
90	10–25	35–64, every 10 years	90	54881858960	19903465960	1710921299	3234807	529
90	10–25	35–64, every 5 years	90	56380415355	19906162723	1498556395	2696763	556
95	10–25	35–64, every 5 years	90	56397275977	19906192323	16860621	29600	570
95	10–25	35–64, every 3 years	76	57432238321	19906753315	1034962344	560991	1845

Table S7b. ICER of strategies that lie on the efficiency frontier compared with next-best strategy for non-vaccinated cohort

Vaccination coverage (%)	Screening	Screening coverage (%)	Discounted costs	Discounted QALY	Incremental costs	Incremental QALY	ICER
95	45	90	37687590520	10335366353	..	..	..
95	35–64, every 10 years	90	40555058508	10340986941	2867467988	5620589	510
95	35–64, every 5 years	90	44569531623	10345485857	4014473115	4498915	892
95	35–64, every 3 years	90	49483675968	10346700340	4914144344	1214483	4046

Table S7c. ICER of strategies that lie on the efficiency frontier compared with next-best strategy for bivalent vaccinated cohort

Vaccination coverage (%)	Screening	Screening coverage (%)	Discounted Costs	Discounted QALY	Incremental costs	Incremental QALY	ICER
95	..	0	6916918430	4509930957	..	..	..
95	45	5	6944874679	4510006753	27956250	75795	369
95	45	10	6972841834	4510082539	27967154	75787	369
95	45	15	7000819890	4510158317	27978056	75778	369
95	45	20	7028808844	4510234087	27988954	75769	369
95	45	25	7056808693	4510309847	27999850	75761	370
95	45	30	7084819435	4510385599	28010742	75752	370
95	45	35	7112841066	4510461343	28021631	75743	370
95	45	40	7140873583	4510537077	28032517	75735	370
95	45	45	7168916984	4510612803	28043400	75726	370
95	45	50	7196971264	4510688520	28054280	75717	371
95	45	55	7225036421	4510764229	28065157	75709	371
95	45	60	7253112453	4510839929	28076031	75700	371
95	45	65	7281199355	4510915620	28086902	75691	371
95	45	70	7309297125	4510991303	28097770	75683	371
95	45	75	7337405759	4511066976	28108635	75674	371
95	45	80	7365525256	4511142642	28119496	75665	372
95	45	85	7393655611	4511218298	28130355	75657	372
95	45	90	7421796822	4511293946	28141211	75648	372
95	40 and 45	90	7954942707	4511675925	533145885	381979	1396
95	40, 45, and 55	90	8526952961	4512078642	572010254	402717	1420
95	40–55, every 5 years	90	9099286240	4512230108	572333279	151466	3779
95	35–64, every 5 years	90	10407014645	4512499772	1307728405	269664	4849
95	35–64, every 3 years	90	12612797057	4512643389	2205782412	143617	15359

Table S7d. ICER of strategies that lie on the efficiency frontier compared with next-best strategy for 9-valent vaccinated cohort

Vaccination coverage (%)	Screening	Screening coverage (%)	Discounted Costs	Discounted QALY	Incremental costs	Incremental QALY	ICER
95	..	0	4060881751	5063738567	..	..	..
95	45	5	4088951052	5063766330	28069301	27763	1011
95	45	10	4117022300	5063794092	28071248	27762	1011
95	45	15	4145095494	5063821852	28073194	27760	1011
95	45	20	4173170635	5063849610	28075141	27758	1011
95	45	25	4201247722	5063877367	28077087	27757	1012
95	45	30	4229326754	5063905122	28079032	27755	1012
95	45	35	4257407732	5063932876	28080978	27754	1012
95	45	40	4285490654	5063960628	28082923	27752	1012
95	45	45	4313575522	5063988379	28084868	27751	1012
95	45	50	4341662334	5064016128	28086812	27749	1012
95	45	55	4369751091	5064043875	28088756	27747	1012
95	45	60	4397841791	5064071621	28090700	27746	1012
95	45	65	4425934435	5064099365	28092644	27744	1013
95	45	70	4454029023	5064127108	28094588	27743	1013
95	45	75	4482125554	5064154849	28096531	27741	1013
95	45	80	4510224028	5064182589	28098474	27740	1013
95	45	85	4538324444	5064210327	28100416	27738	1013
95	45	90	4566426803	5064238063	28102359	27736	1013
95	40, 45, and 55	90	5549846986	5064513384	983420183	275320	3572
95	40–55, every 5 years	90	6022514112	5064569953	472667125	56570	8355
95	35–64, every 5 years	90	7112255101	5064654081	1089740990	84127	12953
95	35–64, every 3 years	90	9003550274	5064701973	1891295172	47892	39490

Table S8. ICERs of switch 9-valent vaccination from 2031 onwards compared to continuing bivalent vaccination*

Vaccination from 2031 onwards	0% discount rate			3% discount rate		
	Total costs million $	Total QALYs million	ICER $/QALY	Total costs million $	Total QALYs million	ICER $/QALY
**China**						
Bivalent	165 285	62 164	..	61 925	19 922	..
9-valent	135 523	62 177	-2468	59 222	19 922	-4077
**Urban area**						
Bivalent	132 999	46 291	..	49 481	14 700	..
9-valent	108 875	46 299	-2748	47 250	14 701	-4519
**Rural area**						
Bivalent	32 286	15 874	..	12 444	5222	..
9-valent	26 648	15 877	-1719	11 972	5222	-2788

ICERs, incremental cost-effectiveness ratios; QALY, quality-adjusted life-year.

* Components may not sum to totals due to rounding.

2. Results

2.1 Sensitivity analysis

Probabilistic sensitivity analyses are displayed in a scatter plot for all discounted/undiscounted incremental costs and quality-adjusted life-years (QALYs) (Figure S4). Each black point on the graph represents one of the incremental costs and the corresponding benefits out of the 1000 simulations.

Univariate sensitivity analyses are displayed in a tornado diagram for all model parameters (Figure S5). In this diagram, each bar represents the impact of uncertainty in an individual variable on the ICER.

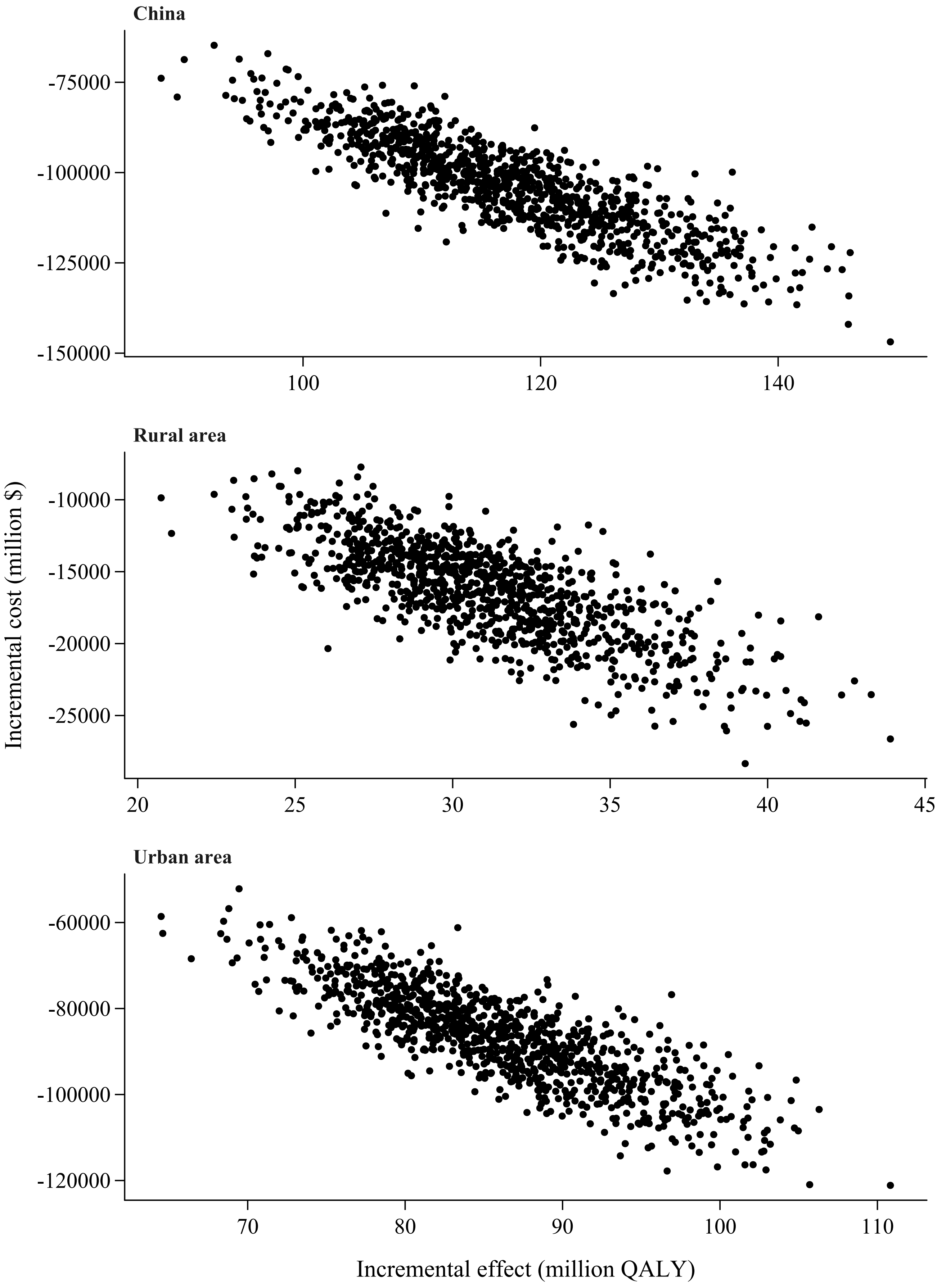

Figure S4a. Probabilistic sensitivity analyses for optimal pathway vs. status quo (0% discount)

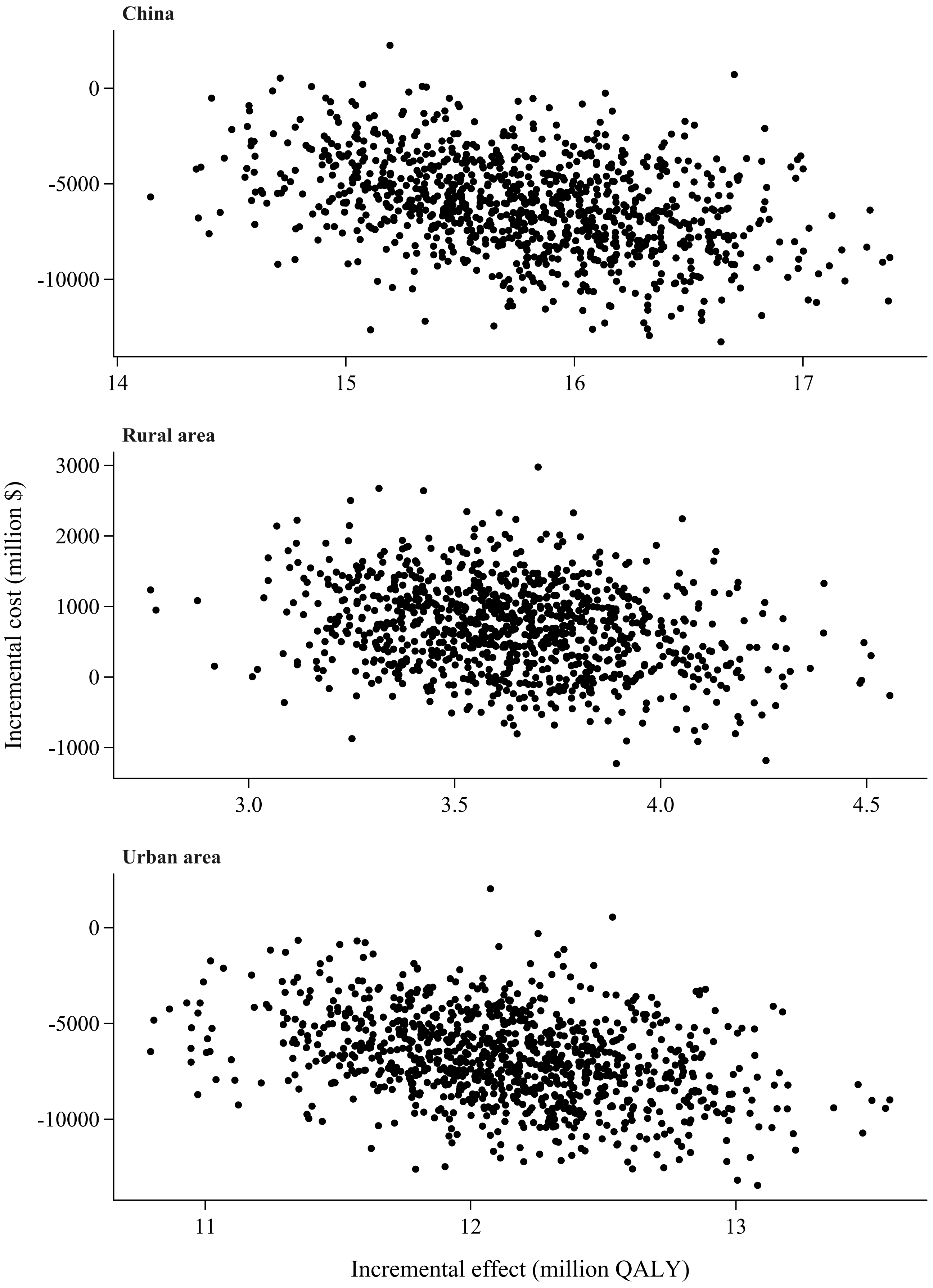

Figure S4b. Probabilistic sensitivity analyses for optimal pathway vs. status quo (3% discount)

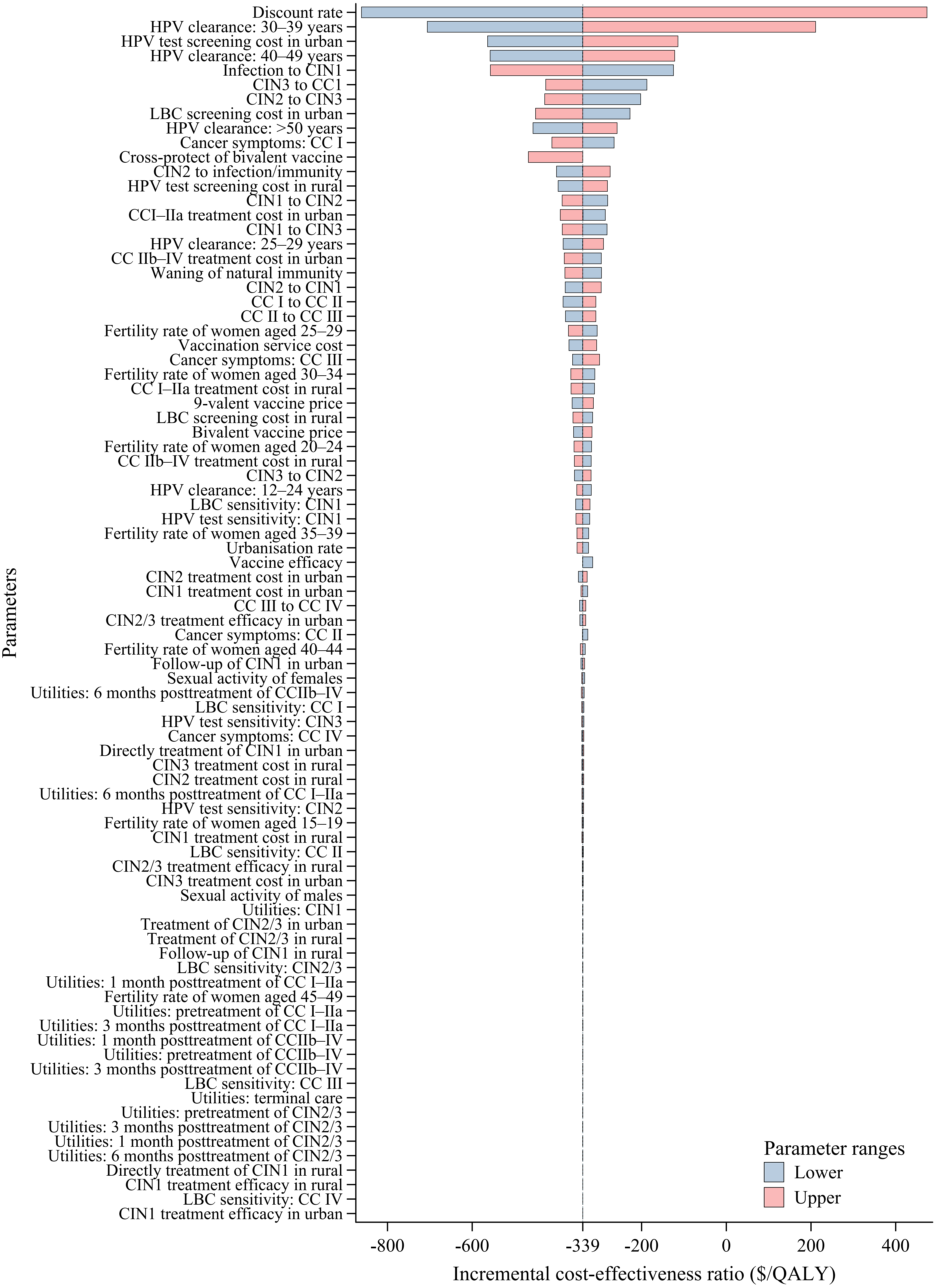

Figure S5a. Deterministic sensitivity analyses for optimal pathway vs. status quo in China

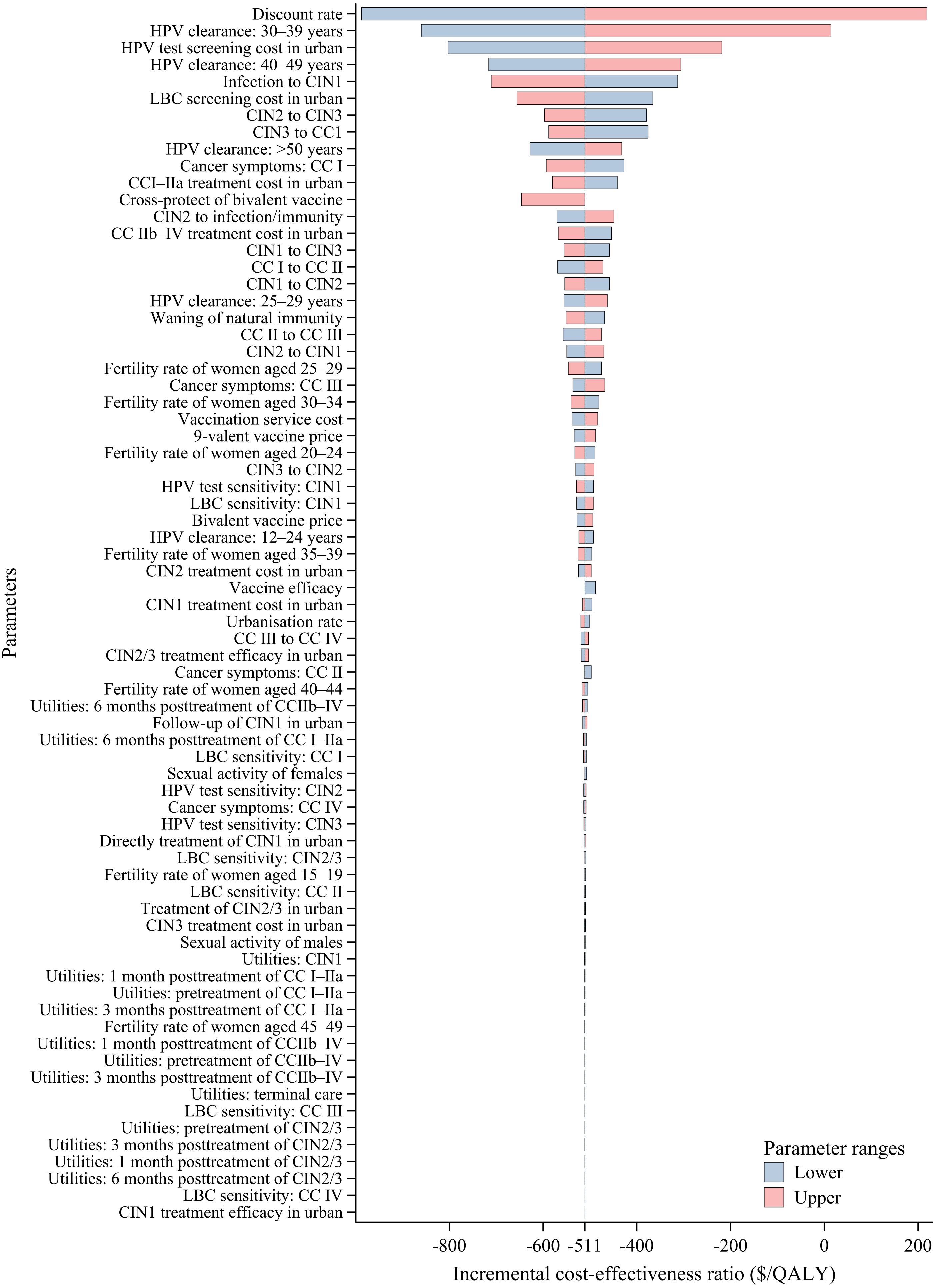

Figure S5b. Deterministic sensitivity analyses for optimal pathway vs. status quo in urban area

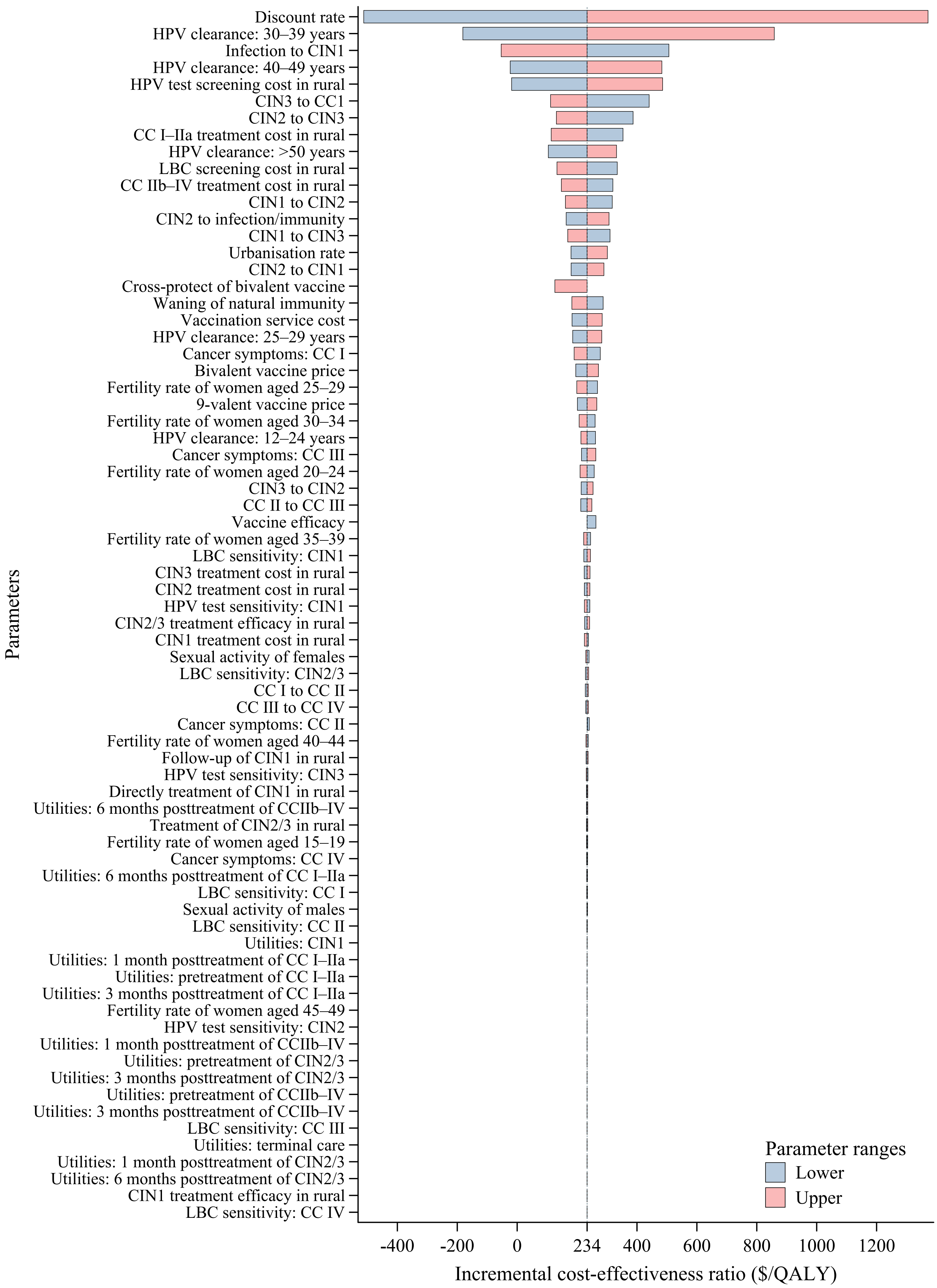

Figure S5c. Deterministic sensitivity analyses for optimal pathway vs. status quo in rural area

2.2 Annual costs and benefits

The annual undiscounted and discounted costs from all four components for optimal pathway and status quo are shown in Figure S6–7. Total annual undiscounted costs for optimal pathway would substantially increase in 2021–25 due to the scale-up of catch-up vaccination and cervical cancer screening, while from 2026 onwards, the total costs of each five years (equal to screening interval of unvaccinated cohorts) would decrease continuously (Figure S6a). Total annual undiscounted costs for status quo would maintain above $1600 million with the variations driven from the transitions of sociodemographic characteristics (Figure S7a). Total annual discounted costs for optimal pathway would be highest in first five years with the peak at 2025, and then substantially decrease to about $53 million in 2100 (Figure S6b). Total annual discounted costs for status quo would substantially decrease to about $156 million in 2100 (Figure S7b).

The annual undiscounted and discounted incremental benefits (ie, QALYs) of optimal pathway compared to status quo are shown in Figure S8. For undiscounted incremental benefits, the values would be negative in first few years before 2026 (filled with red color) because more women with CIN and invasive cancer would be diagnosed, however, values would substantially increase after that and maintain at about one million incremental QALYs from 2070 onwards (Figure S8a). For discounted incremental benefits, although the incremental benefits in the first few years would be negative, the value would always be positive from 2027 onwards with the peak among 2050s (Figure S8b).

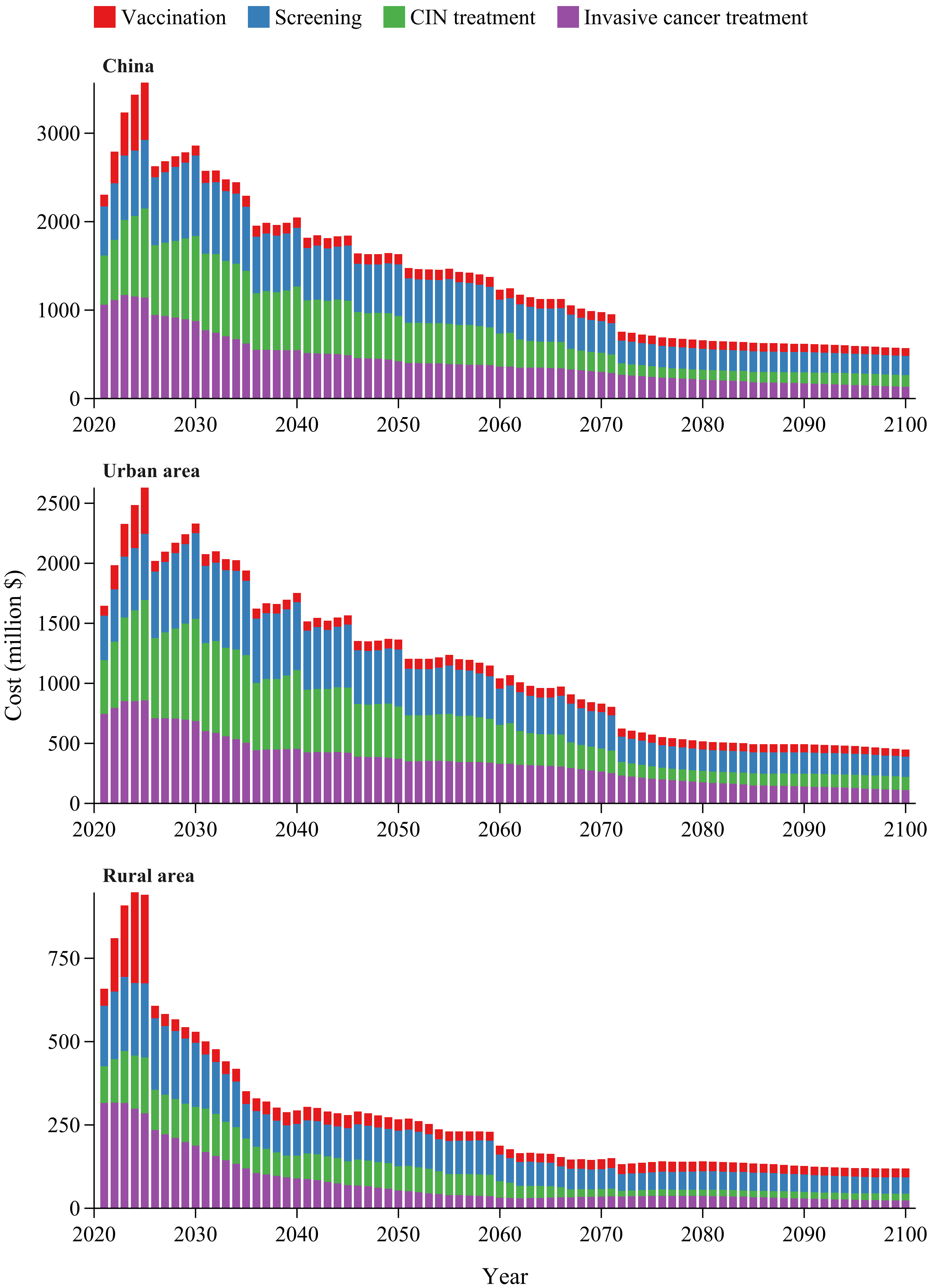

Figure S6a. Annual costs of optimal pathway (0% discount)

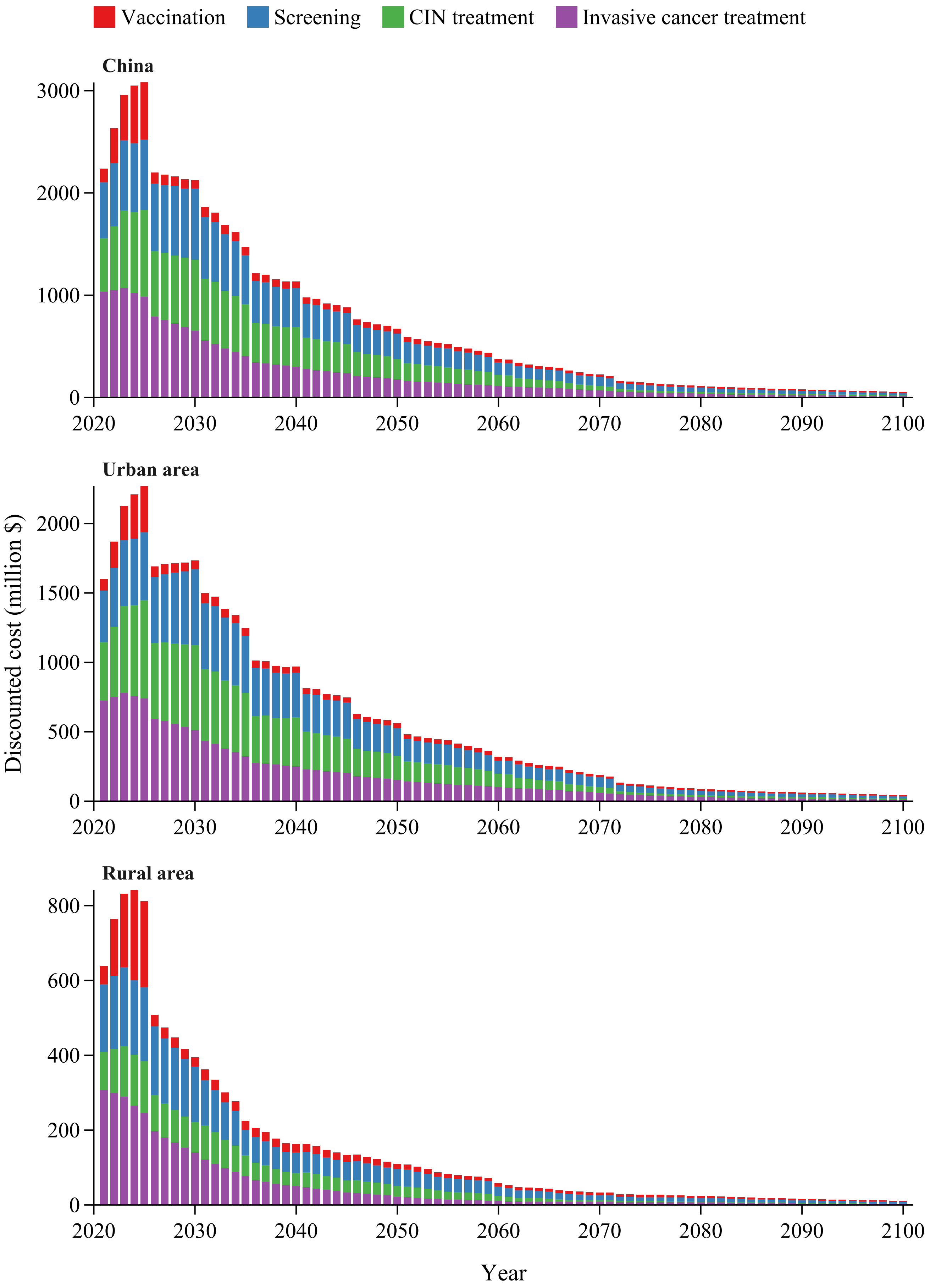

Figure S6b. Annual costs of optimal pathway (3% discount)

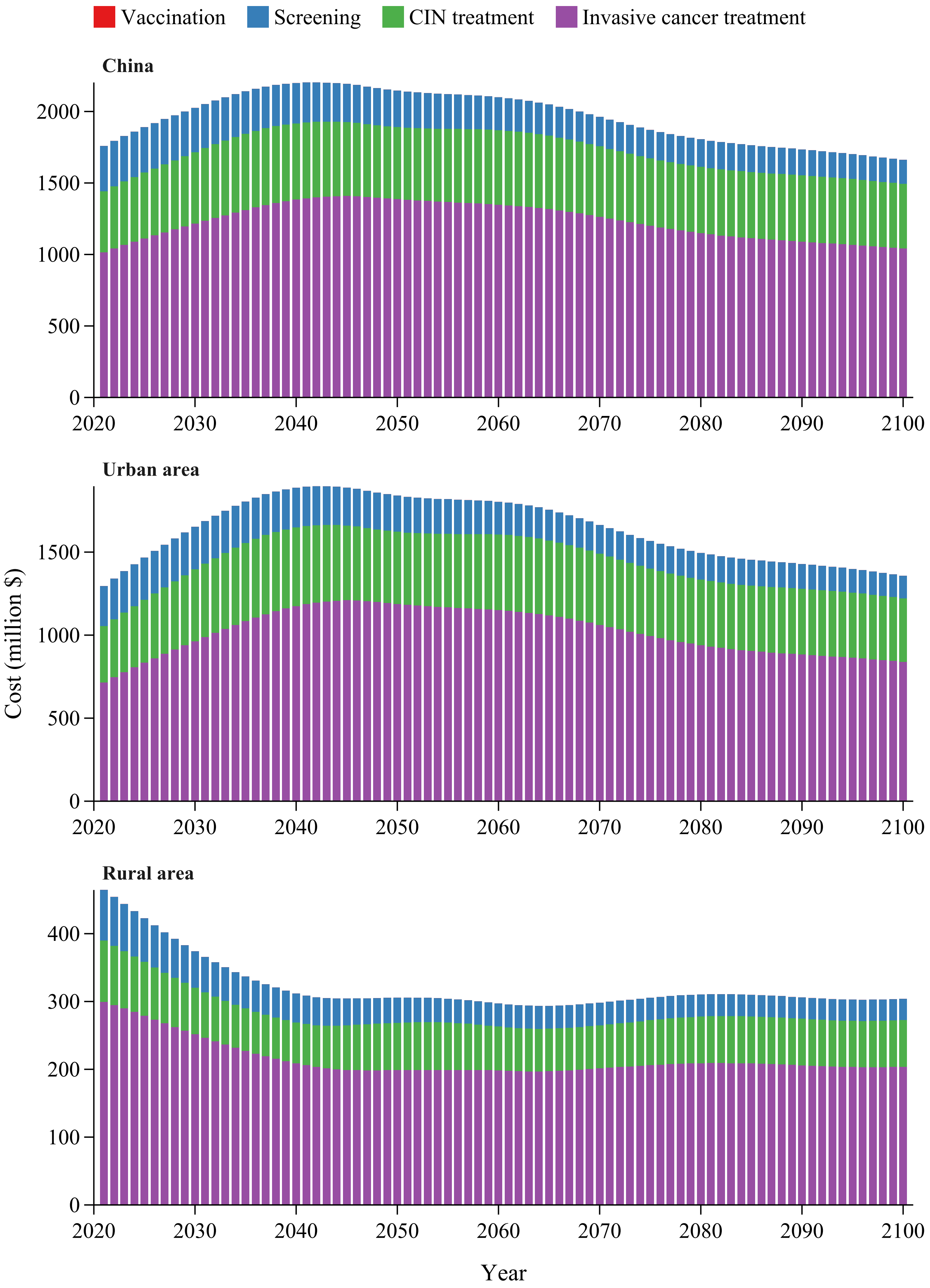

Figure S7a. Annual costs of status quo (0% discount)

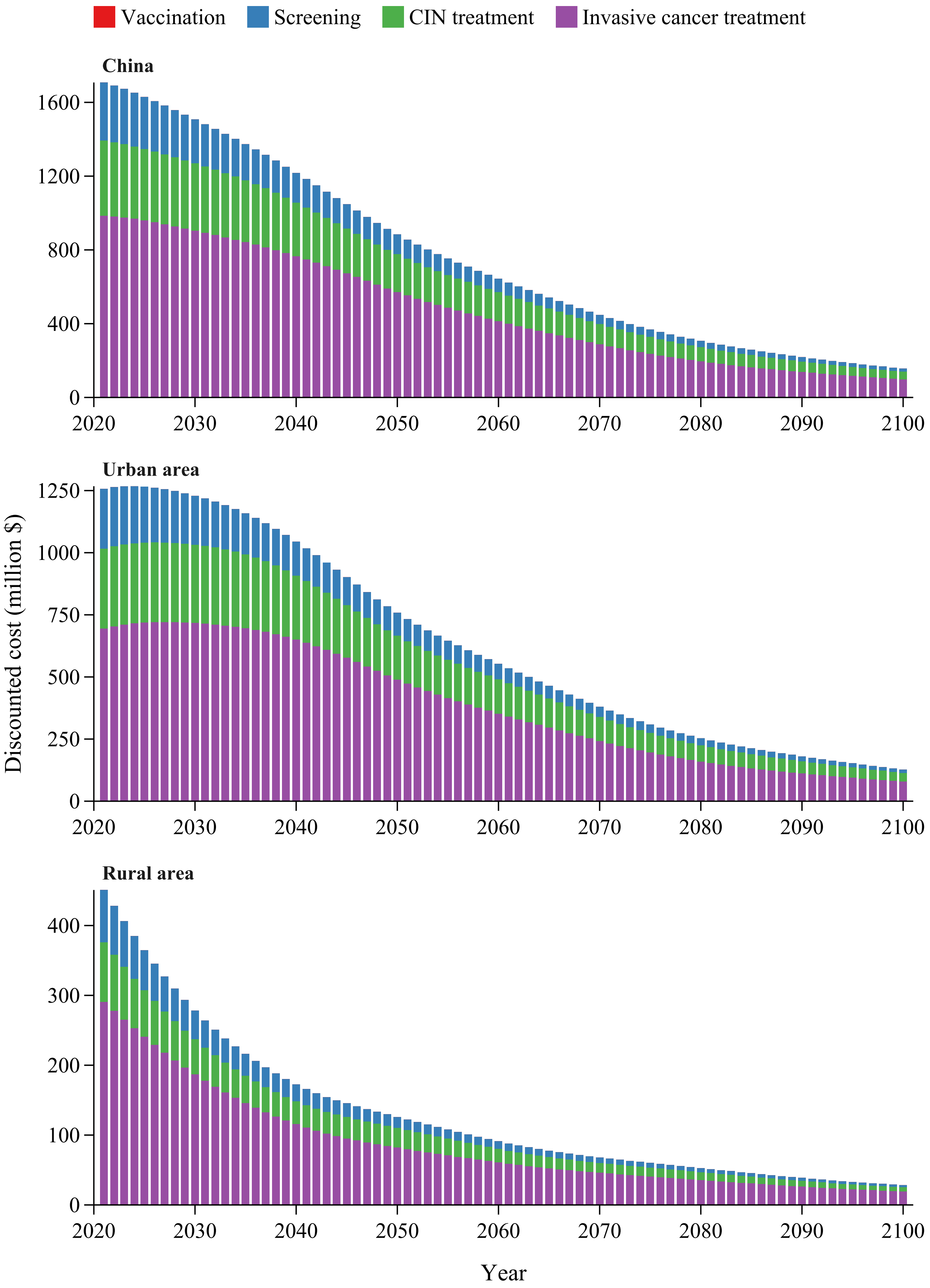

Figure S7b. Annual costs of status quo (3% discount)

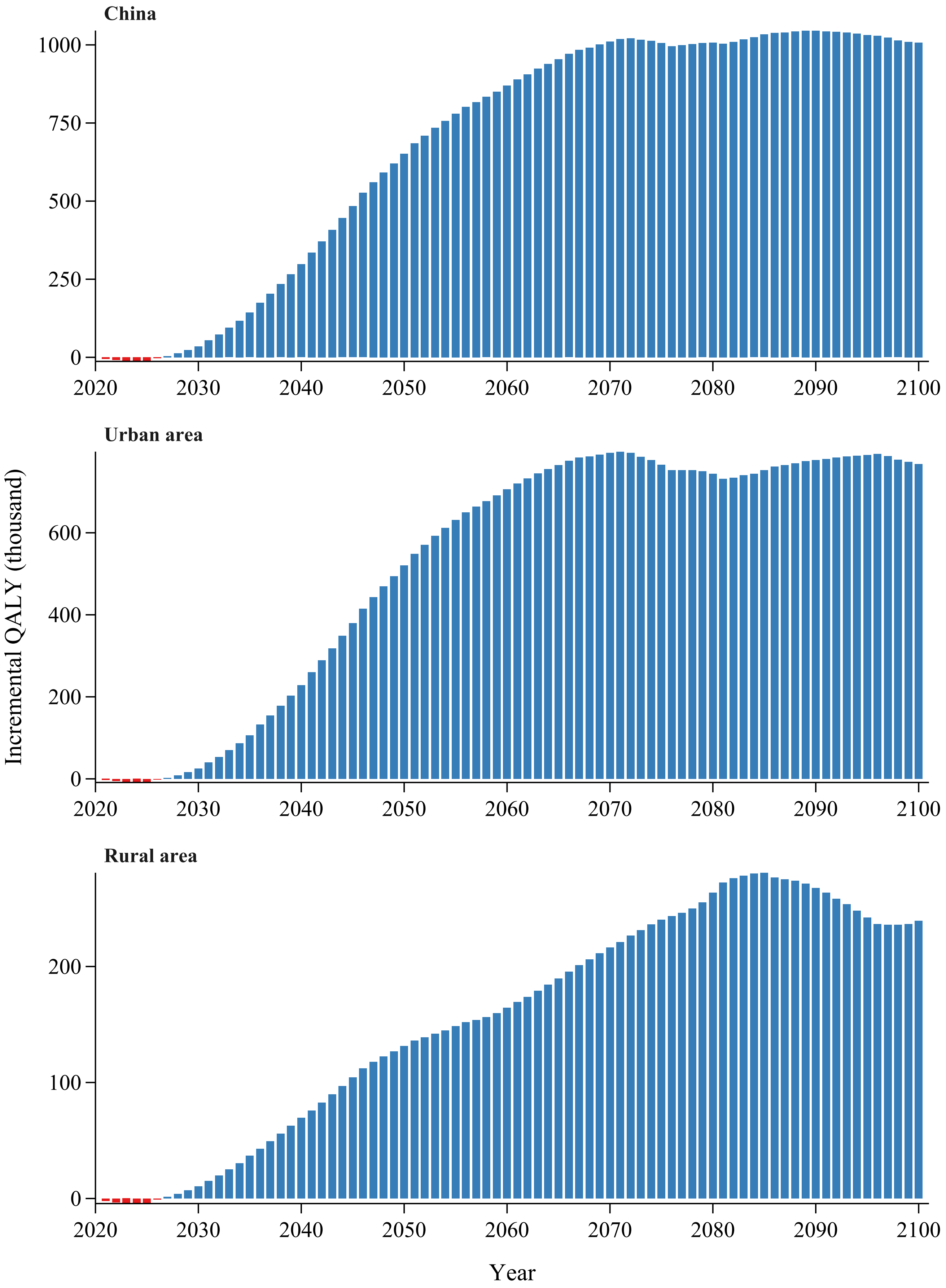

Figure S8a. Annual incremental benefits of optimal pathway compared to status quo (0% discount)

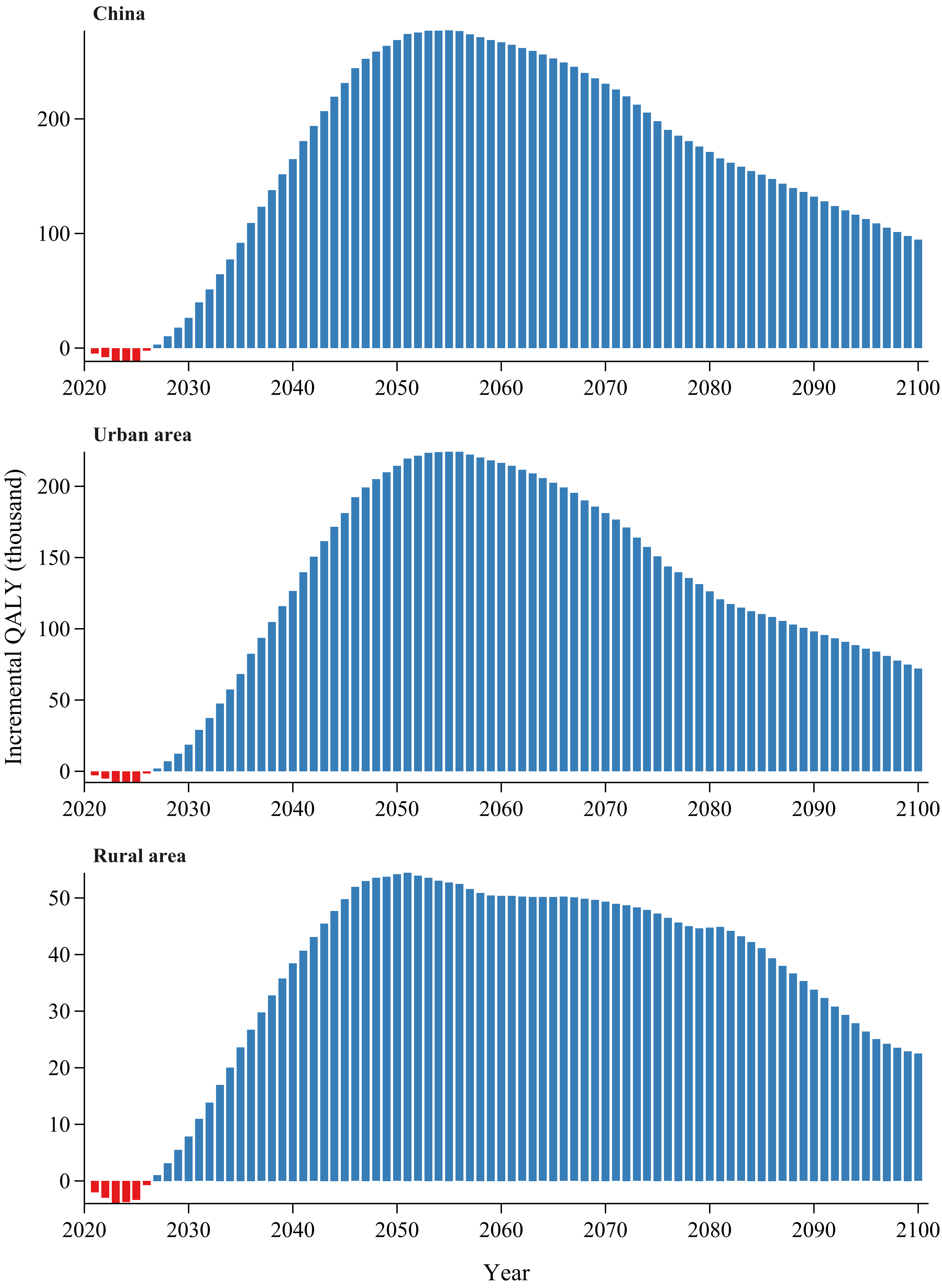

Figure S8b. Annual incremental benefits of optimal pathway compared to status quo (3% discount)

2.3 Use GDP per capita as threshold

2.3.1 Main results

When we use GDP per capita as the threshold, the best strategy simulated by the model was to implement the intensive screening strategy by adopting a shorter screening interval for women compared with the strategy using median annual income as threshold (Figure S9).

- Bivalent HPV vaccination
- 9-valent HPV vaccination
- Screening at 35–64, every 3 years
- Screening at 35–64, every 5 years
- Screening at 40–55, every 5 years
- Treatment status quo for CIN
- Treatment following guidelines for CIN

Age	2021	2022	2023	2024	2025
9	95%	95%	95%	95%	95%
10–13					
14		90%	90%	90%	90%
15		10%			
16		10%	20%		
17		10%	20%	30%	
18–22		10%	20%	30%	30%
23			20%	30%	30%
24				30%	30%
25					30%
35–64	35%	40%	45%	50%	55%

Age	2026	2027	2028	2029	2030
9	95%	95%	95%	95%	95%
10–25					
35–64	60%	65%	70%	73%	76%

Age	2031	2032	2033	2034	2035–61
9	95%	95%	95%	95%	95%
10–25					
35–64 (unvaccinated)	79%	82%	85%	88%	90%
35–64 (bivalent)					90%

Age	2062–81	2082–2100
9	95%	95%
10–25		
35–64 (bivalent)	90%	
35–64 (9-valent)	90%	90%

Note: *The percentages and background colors in each cell indicate the population coverages.*

Figure S9. Optimal pathway towards cervical cancer elimination in China (use GDP per capita as threshold)

HPV, human papillomavirus; CIN, cervical intraepithelial neoplasia.

If China adopts the optimal pathway (use GDP per capita as threshold) from 2021 onwards, cervical cancer would be eliminated by 2035 (2035–2036) for urban area and 2036 (2036–2037) for rural area (Figure S10). The discounted ICER of optimal pathway (use GDP per capita as threshold) compared to status quo were $-62 (-534, 330) for urban area and $566 (160, 939) (Table S9). However, the discounted ICER of optimal pathway using GDP per capita as threshold compared to optimal pathway using median income as threshold were $4305 (3115, 6154) for urban area and $4503 (3386, 6292) (Table S10). In 2021–2100, we estimated that a total of 8312 291 (7710 690, 9075 118) cervical cancer cases and 2863 715 (2701 823, 3089 380) cervical cancer deaths would be averted in China by adopting optimal pathway (use GDP per capita as threshold) from 2021 onwards (Table S11).

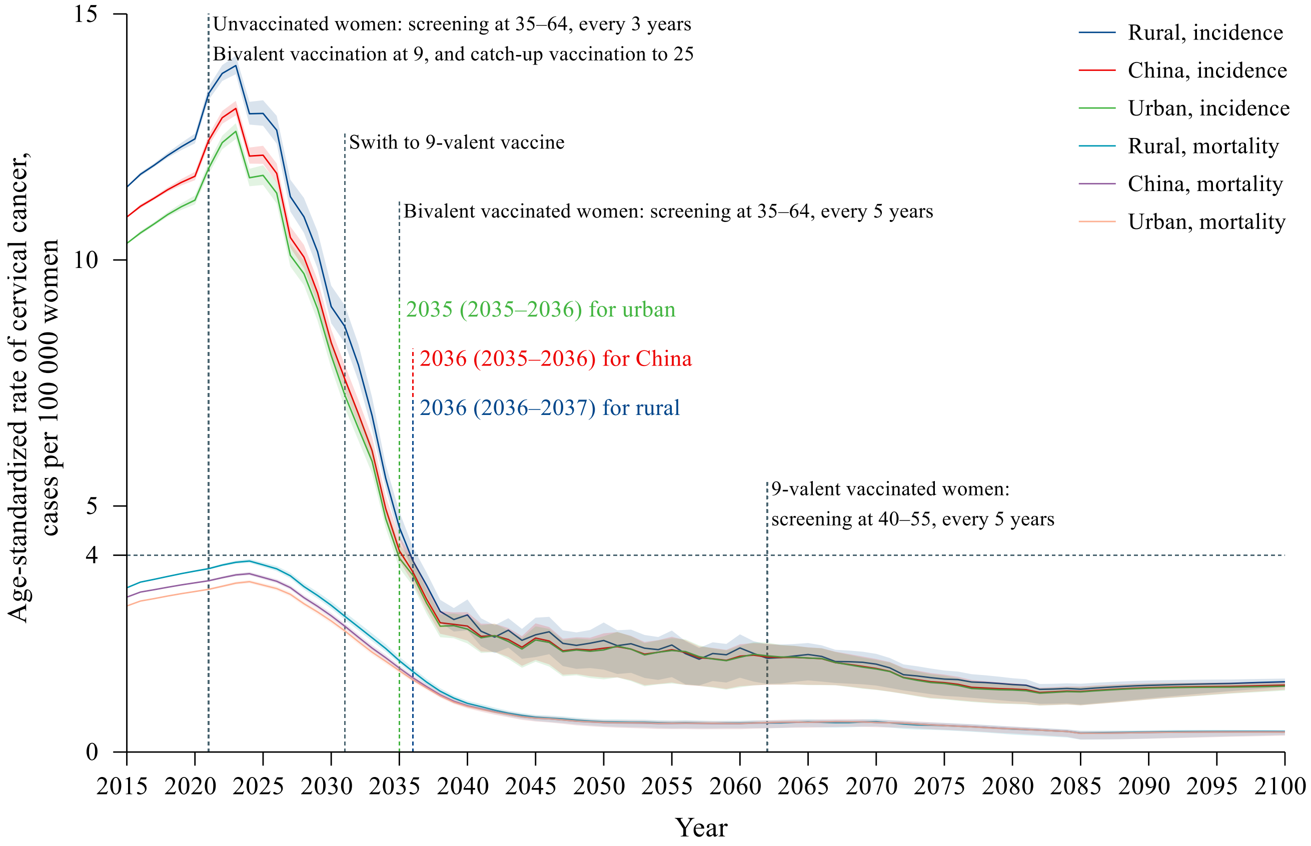

Figure S10. Age-standardized cervical cancer incidence and mortality by adopting optimal pathway (use GDP per capita as threshold)

The solid line represents the base case estimates and shaded area represents the 95% confidence intervals based on 1,000 simulations of probabilistic sensitivity analysis.

Table S9. Lifetime costs, effectiveness, and incremental cost-effectiveness for optimal pathway (use GDP per capita as threshold) vs. status quo*

**Strategy**	**0% discount rate**			**3% discount rate**		
	**Total costs** million $ (95% CI)	**Total QALYs** million (95% CI)	**ICER** $/QALY (95% CI)	**Total costs** million $ (95% CI)	**Total QALYs** million (95% CI)	**ICER** $/QALY (95% CI)
**China**						
Status quo	235 233 (202 975, 274 054)	62 061 (53 812, 71 934)	..	64 516 (60 785, 68 688)	19 907 (18 803, 21 106)	..
Optimal pathway	150 632 (129 921, 172 238)	62 183 (53 917, 72 078)	-691 (-852, -564)	65 916 (58 970, 71 486)	19 924 (18 820, 21 124)	82 (-325, 420)
**Urban area**						
Status quo	192 864 (167 384, 224 928)	46 214 (40 370, 53 409)	..	53 387 (49 723, 57 458)	14 689 (13 864, 15 656)	..
Optimal pathway	120 690 (102 113, 139 856)	46 305 (40 447, 53 515)	-799 (-993, -635)	52 571 (46 353, 58 344)	14 702 (13 877, 15 670)	-62 (-534, 330)
**Rural area**						
Status quo	42 369 (33 919, 53 818)	15 846 (13 269, 19 448)	..	11 130 (9 565, 12 860)	5218 (4733, 5818)	..
Optimal pathway	29 942 (24 469, 35 761)	15 878 (13 295, 19 489)	-388 (-556, -235)	13 346 (11 511, 15 109)	5222 (4737, 5822)	566 (160, 939)

QALY, quality-adjusted life-year; ICER, incremental cost-effectiveness ratio.

* Components may not sum to totals due to rounding.

Table S10. Lifetime costs, effectiveness, and incremental cost-effectiveness for optimal pathway using GDP per capita as threshold vs. optimal pathway using median income as threshold*

Strategy	0% discount rate			3% discount rate		
	**Total costs** million $ (95% CI)	**Total QALYs** million (95% CI)	**ICER** $/QALY (95% CI)	**Total costs** million $ (95% CI)	**Total QALYs** million (95% CI)	**ICER** $/QALY (95% CI)
**China**						
Optimal pathway using median income as threshold	135 523 (117 264, 151 530)	62 177 (53 911, 72 071)	..	59 222 (53 732, 63 311)	19 922 (18 818, 21 122)	..
Optimal pathway using GDP per capita as threshold	150 632 (129 921, 172 238)	62 183 (53 917, 72 078)	2291 (1748, 3377)	65 916 (58 970, 71 486)	19 924 (18 820, 21 124)	4344 (3357, 6056)
**Urban area**						
Optimal pathway using median income as threshold	108 875 (93 177, 123 739)	46 299 (40 442, 53 509)	..	47 250 (42 273, 51 218)	14 701 (13 876, 15 669)	..
Optimal pathway using GDP per capita as threshold	120 690 (102 113, 139 856)	46 305 (40 447, 53 515)	2247 (1597, 3488)	52 571 (46 353, 58 344)	14 702 (13 877, 15 670)	4305 (3115, 6154)
**Rural area**						
Optimal pathway using median income as threshold	26 648 (21 842, 31 565)	15 877 (13 294, 19 488)	..	11 972 (10 405, 13 413)	5222 (4736, 5822)	..
Optimal pathway using GDP per capita as threshold	29 942 (24 469, 35 761)	15 878 (13 295, 19 489)	2462 (1793, 3672)	13 346 (11 511, 15 109)	5222 (4737, 5822)	4503 (3386, 6292)

QALY, quality-adjusted life-year; ICER, incremental cost-effectiveness ratio.

* Components may not sum to totals due to rounding.

Table S11. Estimated cervical cancer cases and deaths averted for optimal pathway (use GDP per capita as threshold) vs. status quo*

Period	Cervical cancer cases averted			Cervical cancer deaths averted		
	**China** no. (95% CI)	**Urban area** no. (95% CI)	**Rural area** no. (95% CI)	**China** no. (95% CI)	**Urban area** no. (95% CI)	**Rural area** no. (95% CI)
2021–30	111 083 (98 742, 123 390)	84 569 (74 722, 94 126)	26 514 (22 126, 30 749)	32 565 (30 981, 34 207)	22 827 (21 443, 24 142)	9 738 (9 021, 10 500)
2031–40	975 672 (943 017, 1009 372)	765 370 (730 500, 800 632)	210 302 (187 132, 233 147)	265 704 (257 398, 274 362)	204 552 (195 316, 213 898)	61 152 (54 495, 67 473)
2041–50	1268 590 (1228 737, 1320 512)	1 016 425 (973 210, 1 067 818)	252 165 (218 342, 288 857)	415 189 (403 243, 429 063)	336 349 (321 605, 352 189)	78 840 (66 820, 91 297)
2051–60	1267 468 (1226 103, 1331 171)	989 151 (945 395, 1 050 744)	278 318 (242 642, 318 430)	442 344 (429 769, 457 883)	357 532 (342 188, 375 361)	84 812 (71 194, 99 286)
2061–70	1211 769 (1152 624, 1294 974)	927 317 (871 790, 999 260)	284 452 (248 300, 327 191)	433 225 (417 722, 453 810)	340 241 (323 270, 359 867)	92 984 (79 542, 107 510)
2071–80	1187 000 (1087 254, 1306 793)	890 458 (809 824, 984 797)	296 542 (257 262, 346 697)	420 658 (394 560, 451 090)	314 831 (292 893, 340 930)	105 827 (92 877, 121 742)
2081–90	1165 217 (1028 059, 1341 754)	861 172 (757 751, 991 408)	304 045 (255 821, 368 804)	433 138 (395 738, 489 460)	319 839 (289 579, 362 590)	113 299 (98 578, 134 674)
2091–2100	1125 492 (946 154, 1347 152)	824 166 (691 958, 989 329)	301 326 (244 812, 378 415)	420 892 (372 412, 499 505)	312 349 (275 049, 372 249)	108 542 (90 588, 134 069)
**Total**	8312 291 (7710 690, 9075 118)	6358 628 (5855 150, 6978 114)	1953 664 (1676 437, 2292 290)	2863 715 (2701 823, 3089 380)	2208 520 (2061 343, 2401 226)	655 194 (563 115, 766 551)

* Components may not sum to totals due to rounding.

2.3.2 Annual costs and benefits

The annual undiscounted and discounted costs from all four components for optimal pathway (use GDP per capita as threshold) are shown in Figure S11. Total annual undiscounted costs would be highest in 2021–35 due to the scale-up of catch-up vaccination and cervical cancer screening, while from 2036 onwards, the total costs of each three years (equal to screening interval of unvaccinated cohorts) would decrease continuously (Figure S11a). Total annual discounted costs would highest in first five years with the peak at 2025, and then decrease to about $59 million in 2100 (Figure S11b).

The annual undiscounted and discounted incremental benefits (QALYs) of optimal pathway (use GDP per capita as threshold) compared to status quo are shown in Figure S12. For undiscounted incremental benefits, the values would be negative in first few years before 2027 (filled with red colour) because more women with CIN and invasive cancer would be diagnosed, however, values would substantially increase after that and maintain at about 1.1 million incremental QALYs from 2070 onwards (Figure S12a). For discounted incremental benefits, although the incremental benefits in the first few years would be negative, the value would always be positive from 2027 onwards with the peak among 2050s (Figure S12b).

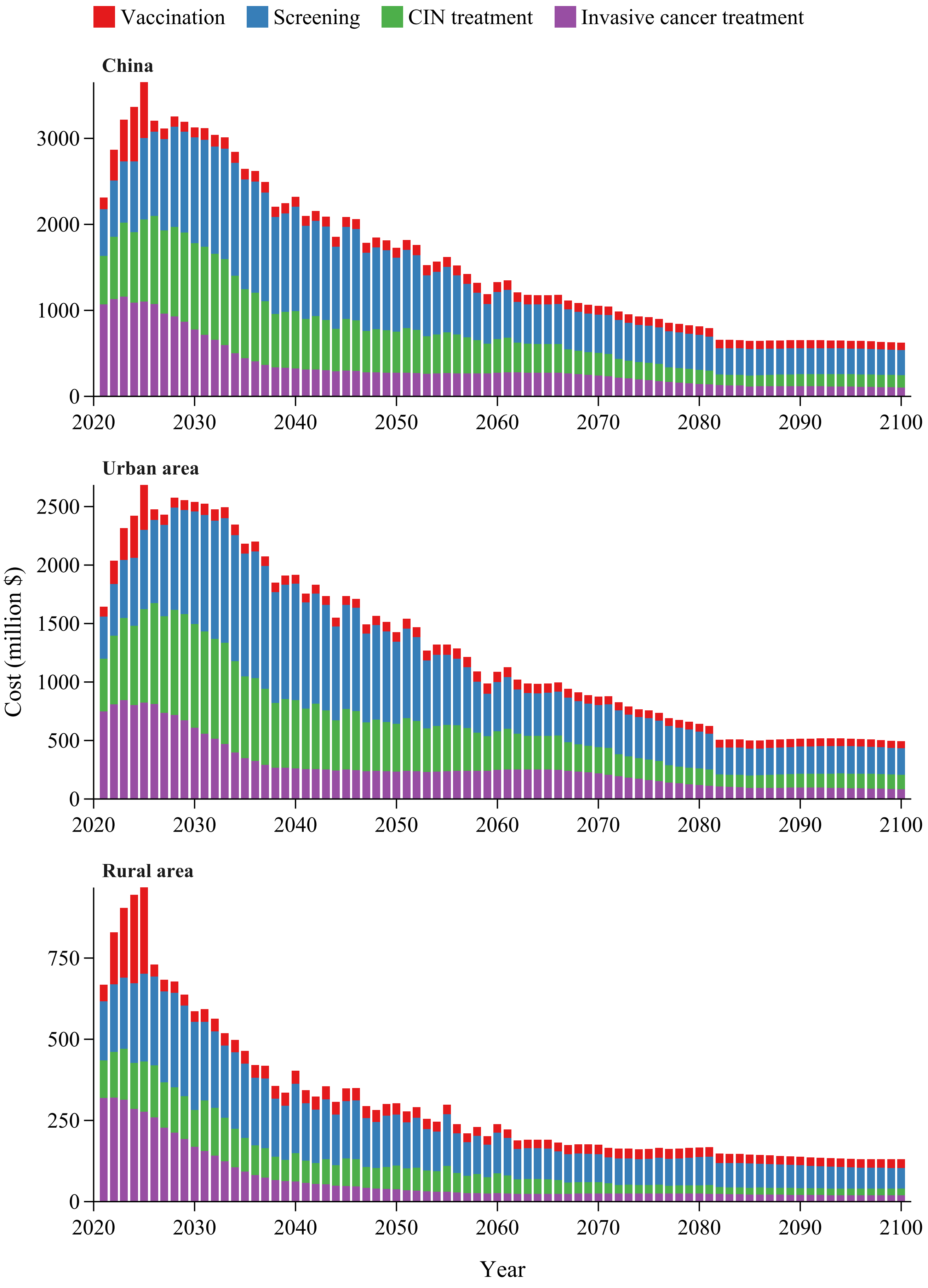

Figure S11a. Annual costs of optimal pathway (use GDP per capita as threshold) (0% discount)

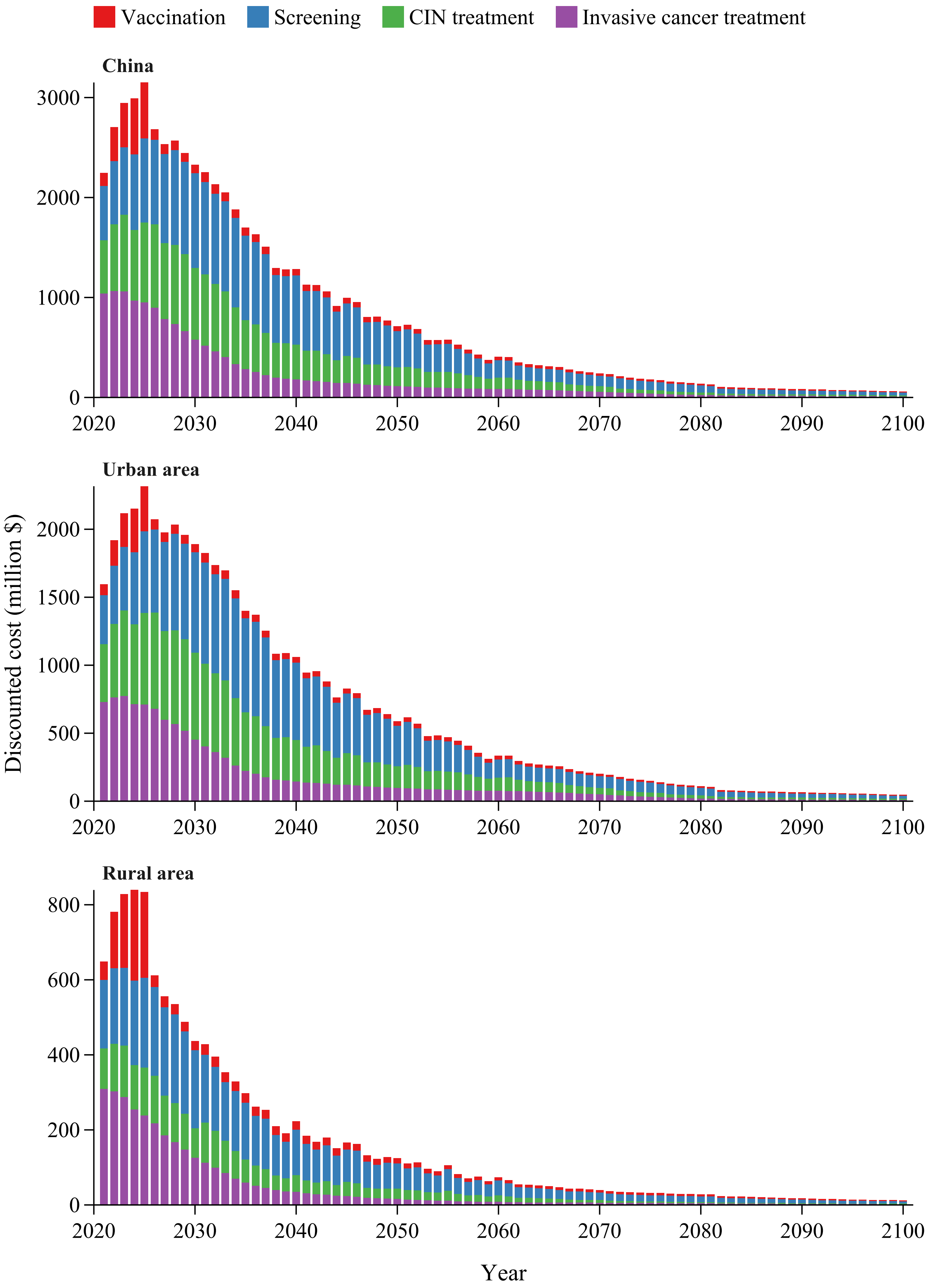

Figure S11b. Annual costs of optimal pathway (use GDP per capita as threshold) (3% discount)

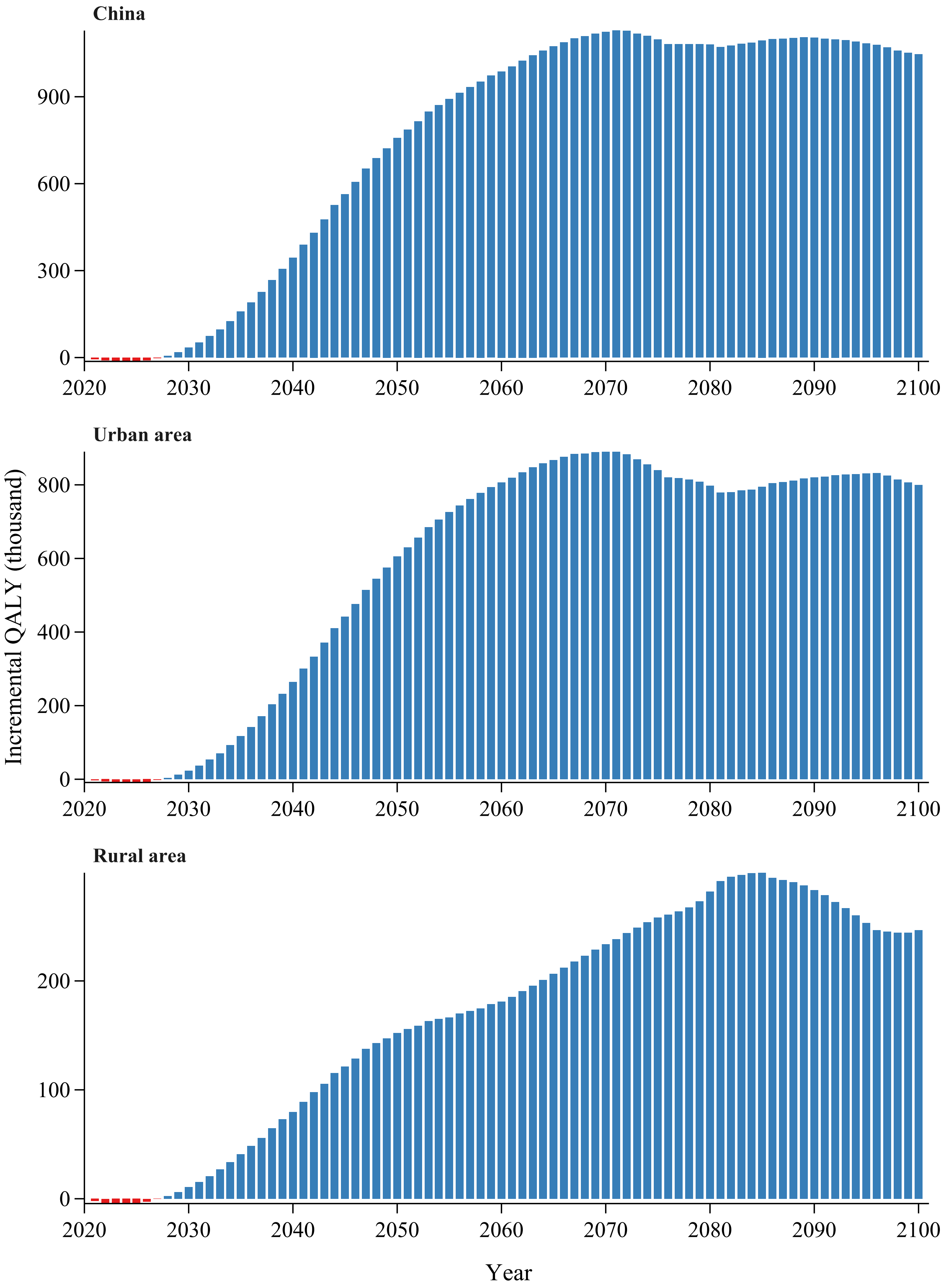

Figure S12a. Annual incremental benefits of optimal pathway (use GDP per capita as threshold) compared to status quo (0% discount)

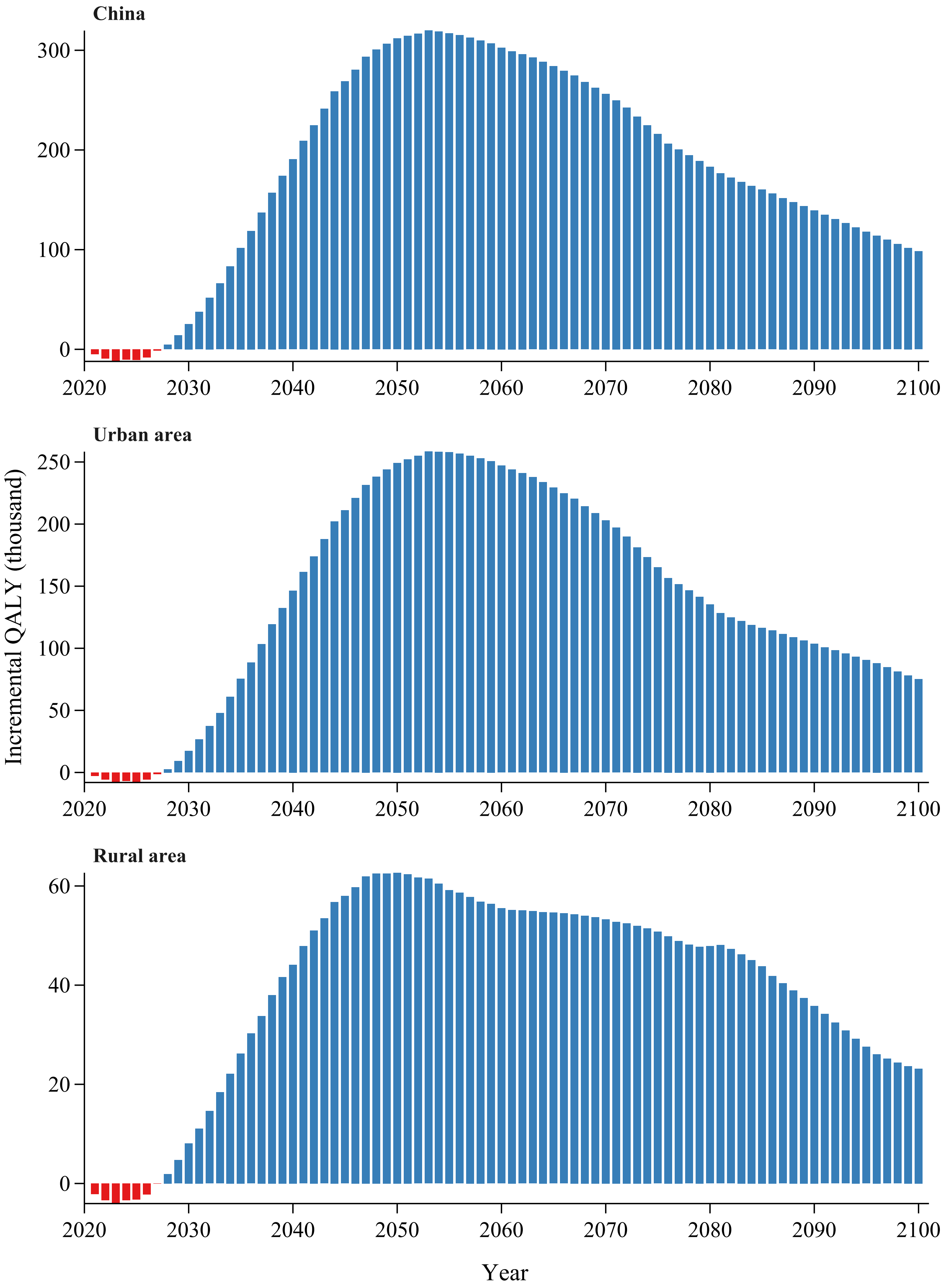

Figure S12b. Annual incremental benefits of optimal pathway (use GDP per capita as threshold) compared to status quo (3% discount)

3. References

1. Xia C, Hu S, Xu X, Zhao X, Qiao Y, Broutet N, et al. Projections up to 2100 and a budget optimisation strategy towards cervical cancer elimination in China: a modelling study. Lancet Public Health 2019;4(9):e462-e72.
2. National Working Committee on Children and Women under State Council. CPPCC National Committee member Yu Luming: Introducing HPV vaccine into the national immunization program. http://www.nwccw.gov.cn/2020-05/22/content_284491.htm. Accessed 6 Jul 2020.
3. Vaccine and Immunization Branch Chinese Preventive Medicine Association. Expert consensus on immunological prevention of human papillomavirus-related diseases. Chinese Journal of Preventive Medicine 2019;53(8):761-803.
4. Zhao F, Qiao Y. Cervical cancer prevention in China: a key to cancer control. Lancet 2019;393(10175):969-70.
5. National Medical Products Administration. First domestic recombinant human papillomavirus vaccine approved. https://www.nmpa.gov.cn/yaowen/ypjgyw/20191231160701608.html. Accessed 6 Aug 2020.
6. Qiao YL, Wu T, Li RC, Hu YM, Wei LH, Li CG, et al. Efficacy, Safety, and Immunogenicity of an Escherichia coli-Produced Bivalent Human Papillomavirus Vaccine: An Interim Analysis of a Randomized Clinical Trial. J Natl Cancer Inst 2020;112(2):145-53.
7. China Global Television Network. China-made HPV vaccine comes to the rescue. https://news.cgtn.com/news/2020-01-09/China-made-HPV-vaccine-comes-to-the-rescue-N7kwyGP9yo/index.html. Accessed 16 Apr 2020.
8. Gavi. HPV vaccine manufacturers commit to provide enough supply to immunise at least 84 million girls in Gavi countries. https://www.gavi.org/news/media-room/hpv-vaccine-manufacturers-commit-provide-enough-supply-immunise-least-84-million. Accessed 7 Jul 2020.
9. Gavi. Gavi-supported HPV vaccines profiles to support country decision making. https://www.gavi.org/sites/default/files/document/2020/Gavi-HPV-vaccine-profiles_May%202020.pdf. Accessed 7 Jul 2020.
10. Yu W, Lu M, Wang H, Rodewald L, Ji S, Ma C, et al. Routine immunization services costs and financing in China, 2015. Vaccine 2018;36(21):3041-7.
11. Centers for Disease Control and Prevention. CDC Vaccine Price List. https://www.cdc.gov/vaccines/programs/vfc/awardees/vaccine-management/price-list/. Accessed 16 Apr 2020.
12. DRUGDATAEXPY. Information of Drug Winning Bid. https://data.yaozh.com/yaopinzhongbiao. Accessed 16 Apr 2020.
13. Beijing Municipal Health Commission. Optimizing and Integrating Screening of Cancers of Cervical and Breast in Beijing: Government Notification. http://wjw.beijing.gov.cn/zwgk_20040/fgwj/gfxwj/201912/t20191216_1239755.html. Accessed 21 Apr 2020.
14. China Hainan Government Procurement. Hainan Women's and Children's Medical Center - HPV testing reagents and related services for women's common diseases and "two cancers" in 2019 -

bid winning announcement. https://www.ccgp-hainan.gov.cn/cgw/cgw_show_zbgg.jsp?id=21831. Accessed 24 Apr 2020.

15. Zhejiang Government Procurement Center. Announcement of bid winning (transaction) results of "two cancer" examination project of provincial Health Commission, HPV and TCT test reagent project. http://zfcg.czt.zj.gov.cn/innerUsed_noticeDetails/index.html?noticeId=6604290. Accessed 24 Apr 2020.
16. Jiangsu Public Resource Trading Platform. TCT and HPV test service project of "two cancers" program in Tongzhou District, Nantong City. http://jsggzy.jszwfw.gov.cn/jyxx/003004/003004002/20200421/f67ce97c-e4f6-44b3-977e-4bc17d5930df.html. Accessed 24 Apr 2020.
17. Peng JR, Tao SY, Wen Y, Yang X, Ma JQ, Zhao F, et al. Cost-effectiveness analysis of cervical cancer screening strategies in urban China. Chinese Journal of Oncology 2019;41(2):154-60.
18. Zhao FH, Lewkowitz AK, Hu SY, Chen F, Li LY, Zhang QM, et al. Prevalence of human papillomavirus and cervical intraepithelial neoplasia in China: a pooled analysis of 17 population-based studies. Int J Cancer 2012;131(12):2929-38.
19. ICO/IARC Information Centre on HPV and Cancer. Human Papillomavirus and Related Diseases in China: HPV Information Centre; 2018.
20. National Bureau of Statistics of China. Residents' income and consumption expenditure in 2019. http://www.stats.gov.cn/tjsj/zxfb/202001/t20200117_1723396.html. Accessed 21 Apr 2020.
21. Tao SY, Peng JR, Wang Y, Zhang GT, Chen ZY, Zhao F, et al. Study on direct economic burden and influencing factors in patients with cervical cancer and precancerous lesions. Chinese Journal of Preventive Medicine 2018;52(12):1281-6.
22. Haeussler KD. A dynamic Bayesian Markov model for health economic evaluations of interventions in infectious disease. London: UCL (University College London); 2017.
23. Haeussler K, den Hout AV, Baio G. A dynamic Bayesian Markov model for health economic evaluations of interventions in infectious disease. BMC Med Res Methodol 2018;18(1):82.
24. Liu YJ, Zhang Q, Hu SY, Zhao FH. Effect of vaccination age on cost-effectiveness of human papillomavirus vaccination against cervical cancer in China. BMC Cancer 2016;16:164.
25. Canfell K, Barnabas R, Patnick J, Beral V. The predicted effect of changes in cervical screening practice in the UK: results from a modelling study. Br J Cancer 2004;91(3):530-6.
26. Goldie SJ, Grima D, Kohli M, Wright TC, Weinstein M, Franco E. A comprehensive natural history model of HPV infection and cervical cancer to estimate the clinical impact of a prophylactic HPV-16/18 vaccine. Int J Cancer 2003;106(6):896-904.
27. Haeussler K, Marcellusi A, Mennini FS, Favato G, Picardo M, Garganese G, et al. Cost-Effectiveness Analysis of Universal Human Papillomavirus Vaccination Using a Dynamic Bayesian Methodology: The BEST II Study. Value Health 2015;18(8):956-68.
28. Myers ER, McCrory DC, Nanda K, Bastian L, Matchar DB. Mathematical model for the natural history of human papillomavirus infection and cervical carcinogenesis. Am J Epidemiol 2000;151(12):1158-71.
29. Yokoyama M, Iwasaka T, Nagata C, Nozawa S, Sekiya S, Hirai Y, et al. Prognostic factors

associated with the clinical outcome of cervical intraepithelial neoplasia: a cohort study in Japan. Cancer Lett 2003;192(2):171-9.

30. Sawaya GF, Sanstead E, Alarid-Escudero F, Smith-McCune K, Gregorich SE, Silverberg MJ, et al. Estimated Quality of Life and Economic Outcomes Associated With 12 Cervical Cancer Screening Strategies: A Cost-effectiveness Analysis. JAMA Intern Med 2019;179(7):867-78.
31. Johnson HC, Elfstrom KM, Edmunds WJ. Inference of type-specific HPV transmissibility, progression and clearance rates: a mathematical modelling approach. PLoS One 2012;7(11):e49614.
32. Brisson M, Kim JJ, Canfell K, Drolet M, Gingras G, Burger EA, et al. Impact of HPV vaccination and cervical screening on cervical cancer elimination: a comparative modelling analysis in 78 low-income and lower-middle-income countries. Lancet 2020;395(10224):575-90.
33. Canfell K, Kim JJ, Brisson M, Keane A, Simms KT, Caruana M, et al. Mortality impact of achieving WHO cervical cancer elimination targets: a comparative modelling analysis in 78 low-income and lower-middle-income countries. Lancet 2020;395(10224):591-603.
34. Abbas KM, van Zandvoort K, Brisson M, Jit M. Effects of updated demography, disability weights, and cervical cancer burden on estimates of human papillomavirus vaccination impact at the global, regional, and national levels: a PRIME modelling study. Lancet Glob Health 2020;8(4):e536-e44.
35. Pan QJ, Hu SY, Zhang X, Ci PW, Zhang WH, Guo HQ, et al. Pooled analysis of the performance of liquid-based cytology in population-based cervical cancer screening studies in China. Cancer Cytopathol 2013;121(9):473-82.
36. Ma L, Wang Y, Gao X, Dai Y, Zhang Y, Wang Z, et al. Economic evaluation of cervical cancer screening strategies in urban China. Chin J Cancer Res 2019;31(6):974-83.
37. van Rosmalen J, de Kok IM, van Ballegooijen M. Cost-effectiveness of cervical cancer screening: cytology versus human papillomavirus DNA testing. BJOG 2012;119(6):699-709.
38. Zhao ZM, Pan XF, Lv SH, Xie Y, Zhang SK, Qiao YL, et al. Quality of life in women with cervical precursor lesions and cancer: a prospective, 6-month, hospital-based study in China. Chin J Cancer 2014;33(7):339-45.
39. Haacker M, Hallett TB, Atun R. On discount rates for economic evaluations in global health. Health Policy Plan 2020;35(1):107-14.
40. National Bureau of Statistics of China. National Data. http://data.stats.gov.cn/english/. Accessed 11 Mar 2020.
41. National Bureau of Statistics of China. China Population and Employment Statistics Yearbook 2016. Beijing: China Statistics Press; 2017.
42. National Health and Family Planning Commission. 2016 China Health Statistics Yearbook. Beijing: Peking Union Medical College Press; 2016.
43. He J. 2018 China Cancer Registry Annual Report. Beijing: People's Medical Publishing House; 2019.
